# Expansion of amphibian intronless interferons revises the paradigm for interferon evolution and functional diversity

**DOI:** 10.1038/srep29072

**Published:** 2016-06-30

**Authors:** Yongming Sang, Qinfang Liu, Jinhwa Lee, Wenjun Ma, D. Scott McVey, Frank Blecha

**Affiliations:** 1 Departments of Anatomy and Physiology, College of Veterinary Medicine, Kansas State University, Manhattan, USA; 2 Diagnostic Medicine and Pathobiology, College of Veterinary Medicine, Kansas State University, Manhattan, USA; 3 Arthropod-Borne Animal Diseases Research Unit, Center for Grain and Animal Health Research, Agricultural Research Service, United States Department of Agriculture, Manhattan, KS, USA

## Abstract

Interferons (IFNs) are key cytokines identified in vertebrates and evolutionary dominance of intronless IFN genes in amniotes is a signature event in IFN evolution. For the first time, we show that the emergence and expansion of intronless IFN genes is evident in amphibians, shown by 24–37 intronless IFN genes in each frog species. Amphibian IFNs represent a molecular complex more complicated than those in other vertebrate species, which revises the established model of IFN evolution to facilitate re-inspection of IFN molecular and functional diversity. We identified these intronless amphibian IFNs and their intron-containing progenitors, and functionally characterized constitutive and inductive expression and antimicrobial roles in infections caused by zoonotic pathogens, such as influenza viruses and *Listeria monocytogenes.* Amphibians, therefore, may serve as overlooked vectors/hosts for zoonotic pathogens, and the amphibian IFN system provides a model to study IFN evolution in molecular and functional diversity in coping with dramatic environmental changes during terrestrial adaption.

Interferons (IFNs) are key cytokines mediating immune and other physiological processes in jawed vertebrates^1^,^2^,^3^. Three types of IFNs (type I, II and III) coexist in tetrapods, distinguished by their difference in molecular signatures, expression patterns, cellular receptors and responsive genes stimulated in various biological processes^3^,^4^,^5^,^6^,^7^. Type I IFNs in amniotes are noted for their single-exon (or intronless) gene structures, which confer evolutionary advantages promoting subtype divergence and expansion through gene copying or duplication. In this context, 10–60 type I IFNs genes have been identified in different amniote species encoding IFNs belonging to at least eight subtypes of IFN-α, -β, -δ, -ε, -κ, -τ, -ω, and -ζ. Some subtypes including IFN-α, -δ, -ω, and -ζ comprise multiple genes in general (IFN-α) or species-specific genes (IFN-δ, -ω, and -ζ)^8^,^9^,^10^,^11^,^12^. Type III IFNs generally consist of 2–4 IFN-λ genes with five exons as reported in most mammalian species^4^,^5^,^12^. In contrast, type II IFN typically has only one member, four-exon IFN-γ, and is conserved in higher animals. Functionally, IFN-γ is mainly produced by activated T-cells and natural killer (NK) cells and is involved primarily in adaptive immunity^1^,^6^,^12^. In contrast, type I and III IFNs, are prominent in rapid induction of innate antiviral defenses bridging adaptive immune responses^2^,^3^,^4^,^5^,^12^. In addition to their general antiviral potency, recent studies indicate that the rapidly evolving antiviral IFN system (including both type I and III IFNs) may functionally multiply through subtype-diversification relative to different expression patterns, antiviral propensity and multifunctional properties involved in other immune and physiological processes^1^,^2^,^3^,^4^,^5^,^13^,^14^,^15^. For example, human IFN-α subtype, in particular IFN-α2, is more effective than IFN-β in antiviral therapies against hepatitis^16^; and human IFN-α11 subtype exerts subtype-specific activation of NK cells to restrict retroviral infections^17^. Recent studies in mice have shown that IFN-ε is specific against viral infections in reproductive tracts^18^, and IFN-λ provides antiviral defense at epithelial surfaces^4^,^5^. In addition to rapid induction during viral infections, constitutive production of IFN-α/β at subthreshold levels is associated with commensal microbiota in regulation of local immune homeostasis^19^,^20^,^21^. Furthermore, some tissue/species-specific IFN subtypes are less relevant to antiviral immunity, such as ruminant IFN-τ and porcine IFN-δ, which are temporally produced in trophoblasts and involved in maternal-fetal recognition during pregnancy^22^,^23^. Collectively, studies of avian and mammalian IFNs imply that the origin of IFN subtypes and functional characteristics is key to understanding IFN evolution and biology^7^,^8^,^9^,^10^,^11^,^12^.

Primitive IFN-like molecules have been identified in fish, with discernible IFN-γ-like genes and IFNϕs^24^. More complex IFN compositions have been characterized in ray-finned fish including zebrafish and salmonids. Zebrafish have two IFN-γ-like genes and four IFNϕs, and there are at least six IFNϕs in salmonids^8^,^24^,^25^. All fish IFN-like genes contain 4–5 exons, and fish IFNϕs could be ancestors of both type I and type III IFNs in amniotes due to their exon-containing (resembling type III IFN genes) or IFNab signature motif (such as CAWE motif preserved at the C-termini of type I IFNs)^8^. Further studies classified fish IFNϕs into two-cysteine and four-cysteine containing groups, and suggested that fish IFNϕs (in particular the four-cysteine group) were more relevant to type I than type III IFNs in amniotes^8^,^9^. Subsequently, several intron-containing type I and type III IFN genes were identified in amphibians. This suggests that type I and III IFNs could have diverged prior to the retroposition process and consequently led to intronless type I IFNs often observed in reptiles, birds and mammals (Fig. 1)^7^,^8^,^9^.

The retroposition process is a reverse-transcription of cellular mRNA and reintegration into the genome, which promotes gene copying and evolution, and is estimated to cause approximately 10,000 gene-duplication events in the human genome^26^. Hence, the origin of intronless IFNs through retroposition is an indicator event in IFN evolution and functional diversification^8^,^9^,^10^,^11^. Previously, intronless IFNs were only identified in type I IFNs in amniotes, and the original retroposition event leading to the emergence of intronless IFNs was assumed to be associated with reptiles (Fig. 1)^7^,^8^,^9^,^10^,^11^,^24^. Here, we report the coexistence of intron-containing and intronless IFNs of both type I and type III IFNs in amphibians, confirming that the original retroposition event leading to intronless IFNs in amniotes actually occurred earlier in amphibians^7^,^8^,^9^,^10^,^11^. Our findings indicate that amphibian IFNs represent an extremely complex IFN system in one species regarding their molecular diversity and functional potency. This discovery not only revises the established model of IFN evolution but also provides a more accurate framework to examine the evolution of IFN-mediated antiviral immunity^8^,^9^. We have functionally characterized these intronless IFNs together with their intron-containing progenitors for their constitutive and inductive expression, and antimicrobial responses in infectious models caused by several zoonotic pathogens^9^,^10^. Amphibians may serve as potential vectors for transboundary and environmental pathogens including influenza and listeria, and the increased diversity of the IFN repertoire in amphibians (versus fish or amniote species) rapidly diversifies their biological activity. These findings demonstrate the critical role of the amphibian IFN system in confronting the unprecedented pathogenic/developmental pressure during their terrestrial adaption, and provide a basis for investigation of IFN molecular evolution and functional diversification in vertebrates.

## Results and Discussion

### Coexistence and expansion of intronless IFNs in amphibians

**Figure 1:**
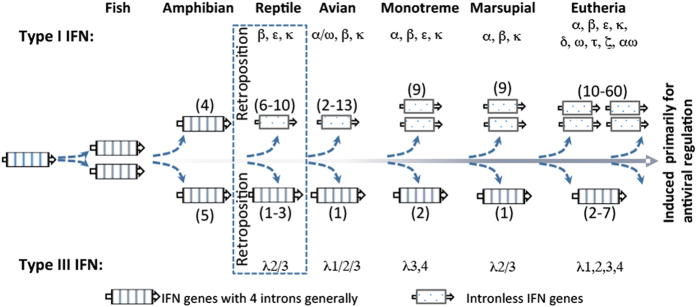
Previous model proposed for the evolution of type I and type III IFNs in vertebrates. In this model, intronless type I IFNs were suggested to first appear in reptiles and diversify linearly in amniotes thereafter. Conversely, type III IFNs maintain highly conserved intron-containing gene structures and family numbers throughout vertebrates^8^.

**Figure 2:**
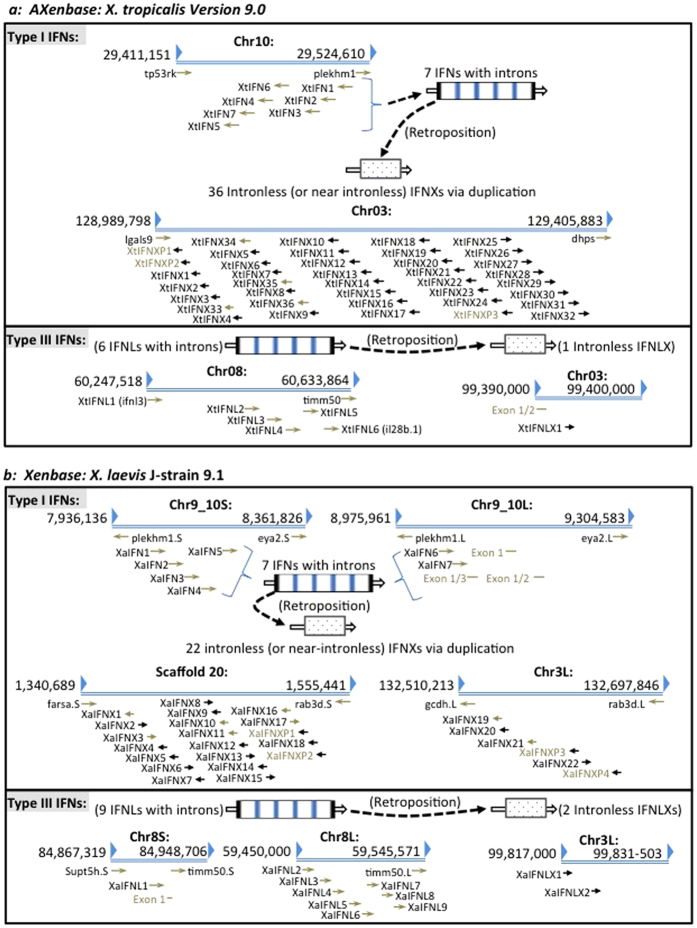
Schematic of types I and III IFN gene loci in amphibians. Schematic distribution of intron-containing and intronless type I and type III IFN genes in chromosomes (or scaffold) in **(a)** *X. tropicalis*, and **(b)** *X. laevis*. The IFN genes were annotated based on three genome assemblies: *X. tropicalis* (*Silurana*) at NCBI (http://www.ncbi.nlm.nih.gov/genome/80, submitted by the DOE Joint Genome Institute), *X. tropicalis* 9.0 genome (*Nigerian*) and *X. leaves* J-strain 9.1 at Xenbase (http://www.xenbase.org/). Some IFN genes and genes bordering IFN loci were identified by automated prediction at Xenbase. The potential retroposition event leading to the emergence of intronless IFNs was depicted to indicate the origin of intronless IFNs from its intron-containing progenitors in amphibians. IFN and IFNX, intron-containing and intronless type I IFNs; IFNL and IFNLX, intron-containing and intronless type III IFNs, respectively.

In the current IFN evolution model^8^, reptiles have been accepted as the segregation point of the origin of intronless IFNs from their intron-containing progenitors in fish and amphibians. Previously, intronless type I IFNs have been observed in higher vertebrates that evolved after reptiles (Fig. 1)^8^,^9^,^10^,^11^. In amphibians, several intron-containing IFNs have been studied in western clawed frogs (*X. tropicalis*)^9^; however, no intronless IFNs have been identified in amphibians^7^,^8^,^9^,^10^,^11^. We have analyzed the current genome assembly of *X. tropicalis* (*Silurana*) at NCBI (http://www.ncbi.nlm.nih.gov/genome/80, submitted by the DOE Joint Genome Institute)^27^, and identified two scaffolds (NW_004668255.1 and NW_004668804.1), which are rich in short regions encoding proteins similar (~50%) to intron-containing frog IFNs (XtIFN) previously identified^9^. Further annotation of the *X. tropicalis* 9.0 genome (*Nigerian*) at Xenbase (http://www.xenbase.org/) identified a ~0.5 Mb region spanning both scaffolds consecutively in Chromosome 3 (Chr03). Through extensive analysis we identified 32 intronless IFN-like genes (XtIFNX), three pseudogenes and four near intronless IFN-like genes retaining only a short (<50 bp) intron. These gene-like sequences could plausibly be rudimentary intermediates of the retroposition event leading to intronless IFN genes. In addition to the first determination of intronless IFNs in frogs, careful annotation of both genome assemblies at NCBI and Xenbase also identified three more intron-containing IFNs clustered in Chr10 and four frog IFN genes previously identified (Fig. 2a)^9^. In the top panel of Fig. 2a, we updated the current repertoire of type I IFN gene loci in *X. tropicalis*: the co-existence of seven intron-containing IFNs and the expansion of nearly 40 intronless IFN-like genes, indicating that the primitive retroposition leading to intron loss of IFN genes and the expansion of resulting single-exon IFN genes may have occurred nearly simultaneously in amphibians rather than later in reptiles^7^,^8^,^9^,^10^,^11^. The emergence of intronless type III IFNs (XtIFNLX) was also investigated within the *X. tropicalis* genome. One single-exon XtIFNLX1 gene encoding a peptide of 209 amino acid residues has considerable protein sequence homology (~50% identity) to previously studied intron-containing type III IFNs that are clustered in Chr08. However, these single-exon IFN genes are located on Chr03 in the *X. tropicalis 9.0 genome* (*Nigerian*), or on a separate Scaffold (NW_004668234.1) of the NCBI *X. tropicalis* (*Silurana*) assembly (Fig. 2a bottom and Supplement Excel Sheet 1).

The coexistence and expansion of intronless IFN-like genes were also verified in tetraploid African clawed frogs (*X. laevis*). Annotation of the genomes of *X. laevis* J-strain (Xenbase) showed similar phenomenon of emergence and expansion of intronless IFNs (Fig. 2b)^28^. Seven intron-containing type I IFNs (XaIFN) were also identified in Chr9-10 of the *X. laevis* J-strain 9.1 genome, and intronless IFN genes (XaIFNX) were clustered in other chromosomes (Scaffold 20 and Chr3L). In total 26 intronless type I IFN genes (including 8 with a short remaining intron and 4 pseudogenes) were identified by both automated prediction (Xenbase) and our manual confirmation^10^. Among the type III IFNs in *X. laevis*, two intronless genes (XaIFNLX) were identified that encode IFN-λ homologous peptides of 183 and 209 residues, respectively (Fig. 2b)^9^. Therefore, the coexistence of intron-containing and intronless IFNs was identified in amphibians^9^. This indicates that the retroposition process leading to the dominance of intronless IFNs in higher amniotes, actually originated in amphibians and not in reptiles as previously believed (Fig. 1)^7^,^8^,^9^,^10^,^11^. The near simultaneous expansion of intronless type I IFNs after emerging in amphibians, implies a potential evolutionary advantage of these single-exon IFN genes in responding to infectious and developmental pressures faced during adaption to terrestrial lives^7^,^8^,^9^,^10^,^11^. Duplicated genes can arise via various mechanisms — polyploidization, chromosomal duplication, segmental duplication, and retroposition^26^. As the major expansion of intronless IFNXs in both frog species was promoted by retroposition, the further selection of IFNXs in amphibians likely resulted from other mechanisms such as polyploidization and chromosomal duplication^29^,^30^. Polyploidization increases cellular and genetic complexity, which could promote better adaptation to chronic injury or stress. However, during evolution of polyploid orders such as plants and animals, polyploidization and consequent ploidy reversal actually led to a high rate (50–75%) of loss of duplicated genes in teleost fish and plants, and approximately 17% loss of duplicated genes in tetraploid *X. laevis* as compared with diploid *X. tropicalis*^29^,^30^. In this context, the 26 intronless IFNX genes in *X. laevis* might represent a less redundant repertoire than the 40 genes in *X. tropicalis*. Compared with the expansion of type I IFNs after the emergence of intronless IFN genes in amniotes, it is not clear why the composition of type III IFNs continued to be relatively stable. The coexistence of intronless type I and type III IFNs in amphibians that evolved distinctly, provides a unique model to study the genetic mechanisms in regulation of IFN molecular and functional diversification^7^,^8^,^9^,^10^,^11^. Interestingly, both intronless type I and III IFN gene loci distribute in the same frog chromosome (Chr03) separated only by an approximate 30-Mb interval (Fig. 2a,b).

### Accelerated subtype divergence in intronless IFNs demonstrated by phylogenetic analyses

**Figure 3:**
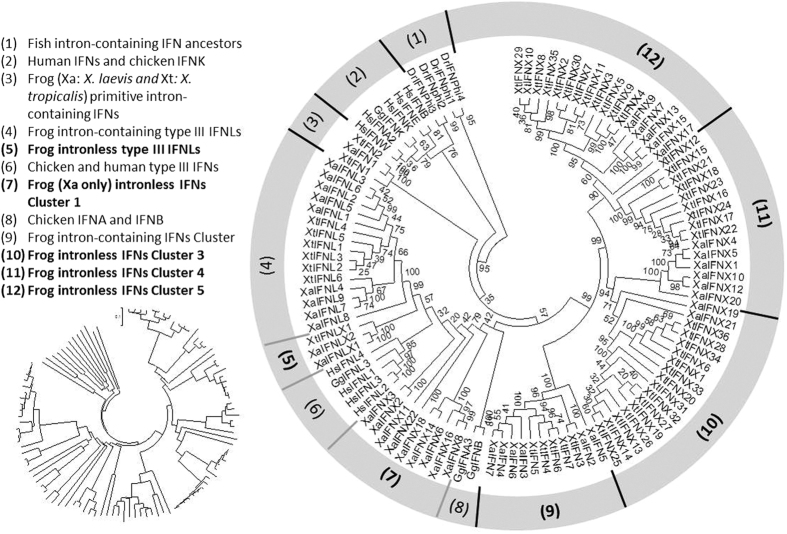
Evolutionary relationships of the IFN complex in *X. laevis* (XaIFNs) and *X. tropicalis* (XtIFNs), and comparison with homologs from zebrafish (DrIFNs), chicken (GgIFNs) and humans (HsIFNs). The evolutionary history was inferred using the Neighbor-Joining method^31^. Percentage of replicate trees in which the associated taxa clustered together in the bootstrap test (1000 replicates) is shown next to the branches. The tree is drawn to scale, with branch lengths in the same units as those of the evolutionary distances used to infer the phylogenetic tree. The evolutionary distances were computed using the p-distance method and are in units of the number of amino acid differences per site. Evolutionary analyses were conducted in MEGA6^32^. The 12 clades/clusters of IFNs, including primarily 5 Clusters of intronless IFNs in amphibians, are listed on the left, and shown with bootstrap values in the main topological tree. The inlet phylogenetic tree at the bottom reflects the relative distance of each leaf. IFN and IFNX, intron-containing and intronless type I IFNs; IFNL and IFNLX, intron-containing and intronless type III IFNs, respectively. IFN taxa used: IFNA, IFNB, IFNE, IFNK, IFNL, and IFNW correspond to genes for IFN-α, IFN-β, IFN-ε, IFN-κ, IFN-λ, and IFN-ω, respectively, in classic nomenclature, and stand for relevant IFN protein precursors here.

We have performed phylogenetic analyses of frog IFNs using gene sequences, cDNA sequences, and primary protein sequences with homologs from fish, chickens, and humans^10^. Figure 3 shows the evolutionary relationship of these IFN peptides inferred from Neighbor-Joining method analysis^31^,^32^. In general, with four fish IFNs rooting the phylogenetic tree of these IFNs, the selected human type I IFNs and chicken IFNK form into one clade separated from amphibian IFNs, indicating a closer relation among them than with frog IFN homologs^7^,^8^,^9^,^10^,^11^. Three amphibian intron-containing IFNs (XaIFN1, XtIFN1, and XtIFN2) appear to be evolutionary links between fish and tetrapod IFNs, and also serve as progenitors for IFN evolution and the emergence of intronless IFNs in amphibians. Both human and chicken type III IFNs (HsIFNLs and GgIFNLs, respectively) annex the cluster of amphibian type III IFNs^9^; and the clade of three frog intronless IFNLXs position between the clades of intron-containing IFNLs of frogs and birds/humans, indicating evolutionary bifurcation of intronless IFNLXs in amphibians from intron-containing type III IFNs in amniotes. The emergence and expansion of intronless IFNs in amphibians could have occurred within a time scale close to the evolution of avian type I IFNs as indicated by the clade of chicken IFNA/B annexing frog intronless IFN Cluster 1 (Group 7 in Fig. 3). In general, all type I intronless IFNs in frogs form into four clusters (Group 7, and 10–12 in Fig. 3), with Cluster 1 comprising only XaIFNXs from tetraploid *X. laevis*, likely implying more diverse intronless IFNs after polyploidization. All other Clusters 9–12 of frog IFNs contain both XaIFNs and XtIFNs. In this regard, most sub-clades of frog IFNs have a XaIFN progenitor, such as XaIFN5, XaIFNX19, XaIFNX20, and XaIFNX17 for IFN Cluster 9, 10, 11, and 12, respectively, in the phylogenetic tree (Fig. 3). It is unexpected that the polyploid *X. laevis* harbors fewer but more conserved intronless IFNs than in the diploid *X. tropicalis.* This, however, is consistent with recent discoveries characterizing polyploidy in amphibia^29^,^30^. Unlike in fish and amphibians, there is no known polyploid mammalian species; however, programmed polyploidization occurs in selected tissues or cells in all mammalian species. For examples, up to 50% or 90% hepatocytes are polyploid in human or mouse livers, respectively^33^. It has been shown that hepatocyte polyploidy renders cells more resistant to apoptosis and more susceptible to parasitic infection^33^,^34^. Therefore, it is tempting to consider if polyploidy in mammalian cells compromises the immune gene repertoire, or if polyploid frogs are more susceptible to some infections due to genomic instability^35^,^36^.

### Retrotransposons associated with amphibian IFNs are identified within primitive IFNX genes

To determine what retrotransposons were potentially involved in the retroposition event leading to the origin of intronless IFN genes, we identified repetitive elements and examined retrotransposons especially within the genomic segments spanning both intron-containing and intronless IFN genes^10^. Some types of retrotransposons are shown to be especially associated with XaIFNX genes, including XaIFNX19–22 that are apparently more primitive than other amphibian IFNX genes (Table 1 and Supplemental Excel Sheet 2, and context associated with Fig. 3 above). This region in Chr03 of *X. laevis* (Fig. 2b), which harbors the insertion/expansion of amphibian intronless IFN genes, contains a significant higher proportion of retrotransposons (15%). In particular, the precursor protein of XaIFNX22 contains an N-terminal 56 residues resembling IFNs, but its C-terminal segment (152 aa) has near 50% similarity to a reverse transcriptase derived from a retrotransposon (Table 1). This may provide a mechanism to identify the original event of retroposition that initialized intronless IFN gene insertions in amniotes. Other amphibian IFN loci, particularly the genomic region spanning the intronless IFN locus of diploid *X. tropicalis,* contain retrotransposons that account for 6% to 9% of the genomic location. However, ERV type of retrotransposons and hAT type of DNA transposons are associated with most ancestral XtIFNX genes, including XtIFNX1, X2/7, X11, and X12. They are the most likely genomic elements associated with intronless IFN gene copying and expansion in the *X. tropicalis* genome (Supplemental Excel Sheet 3)^8^,^9^,^10^,^11^.

### Dominant and directional positive selection of intronless IFNX genes in *X. tropicalis*

**Figure 4:**
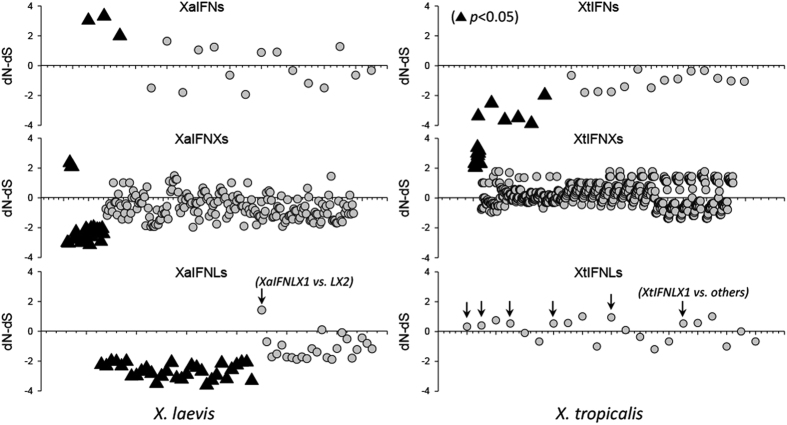
Codon-based test of neutrality for analysis between sequences of amphibian IFN coding regions. The dS and dN are numbers of synonymous and nonsynonymous substitutions per site, respectively. Probabilities of rejecting the null hypothesis of strict-neutrality (dN = dS, middle zero lines) is shown. Values of *p* less than 0.05 are considered significant at the 5% level and are highlighted (black triangles). The test statistic (dN–dS) is shown on Y-axis. Symbols below the dN = dS line are considered in negative selection (or purifying selection) and above the zero lines, positive selection. Analyses were conducted using the Nei-Gojobori method and evolutionary analyses were conducted in MEGA6^32^. Results suggest that an eventual functional transition occurred from the coexisting intron-containing IFNs to the newly emerged and more effective intronless antiviral IFNs in amphibians, showing that significant positive selections occurred in both intron-containing (left top) and intronless (left middle) type I IFNs in *X. laevis*, but only were detected in intronless IFNX genes in *X. tropicalis* (right middle). In addition, most cases of positive selection were detected between the sequence pairs containing one or two intronless type III IFNs (indicated by arrows in the bottom two plots, and Supplemental Excel sheet 4). IFN and IFNX, intron-containing and intronless type I IFNs; IFNL and IFNLX, intron-containing and intronless type III IFNs, respectively.

We performed codon-based analysis of natural selection among coding sequences within each sub-group of amphibian IFNs: intron-containing and intronless type I IFNs, and type III IFNs in either diploid or tetraploid frogs (Fig. 4, and Supplemental Excel Sheet 4)^32^,^37^. The probability of rejecting the null hypothesis of strict-neutrality is shown with the middle zero lines indicating dN = dS (dS and dN are the numbers of synonymous and nonsynonymous substitutions per site, respectively). Hence, the black-triangle symbols above or below the zero lines in each plot indicate compared sequence pairs that have significant (p < 0.05) positive or purifying selection, respectively; and grey circles show non-significant selections between IFN coding sequences in each subgroup. In *X. laevis* (Fig. 4, left panel), significant positive selection was detected between several pairs of intron-containing (XaIFNs) and intronless type I IFNs (XaIFNXs). However, significant purifying selection was more prominent in subgroups of both intronless type I IFNs (XaIFNXs) and type III IFNs including two intronless XaIFNLXs. In *X. tropicalis* (Fig. 4, right panel), significant positive selections were only detected between intronless type I IFNs (XtIFNXs), and intron-containing type I IFNs (XtIFNs) undergoing significant purifying selection. Together, most subgroups of amphibian IFNs may be subject to bidirectional selection below the statistically significant threshold. However, type III IFNs of *X. laevis* (XaIFNLs) and intron-containing type I IFNs of *X. tropicalis* (XtIFNs) did undergo predominant purifying selection. These data suggest an eventual functional transition from the coexistence of intron-containing IFNs to the newly emerged intronless antiviral IFNs in amphibians. In *X. laevis*, the active natural selection in intronless IFNs was accompanied with the intron-containing type I IFNs (XaIFNs). Both groups of type I IFNs (intron-containing and intronless) appeared to function well to experience actively natural and particularly positive selections towards advantageous genetic variants. However, the intron-containing IFNs in *X. tropicalis* experienced only purification selection, relinquishing to the expanding intronless ones. It is notable that most cases of positive selection (indicated by arrows in the bottom two plots of Fig. 4, and Supplemental Excel Sheet 4) were detected between sequence pairs containing one or two intronless type III IFNs of *X. tropicalis* or X. *laevis,* respectively. This potentially indicates the evolving efficacy of intronless IFNs to outcompete their intron-containing counterparts. However, it is unclear why intronless type III IFNs eventually lost this evolving superiority in most amniote species, which is probably due to the evolutionary parsimony resulting from functional redundancy of the coexisting intronless type I IFNs^7^,^8^,^9^,^10^,^11^. The relatively conserved nature of intron-containing IFNs may function to better maintain immune and physiological homeostasis of the mucosal epithelia, where type III IFNs are preferentially expressed and functional^4^,^5^.

### Molecular and functional subgroups of the amphibian IFN complex

**Figure 5:**
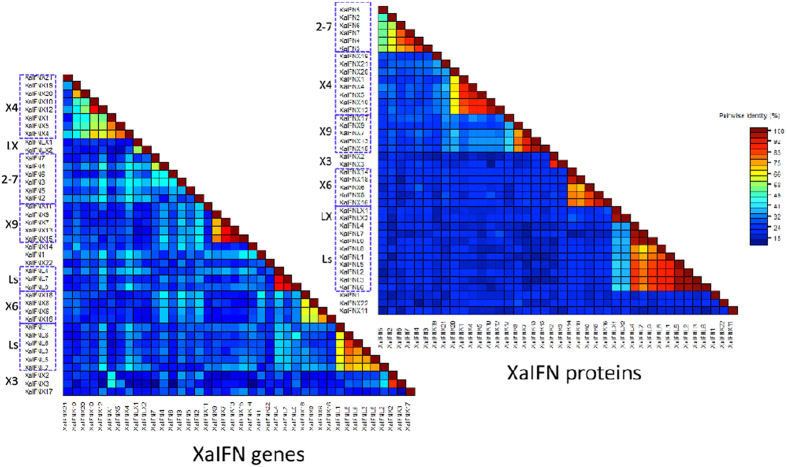
Pairwise identity (%) plots among protein (top right) and gene (bottom left) sequences of IFNs in *X. laevis*. Comparison and plot drawing were performed using a SDT program. Molecular subgroups of IFN complex in *X. laevis* were clustered based on sequence identity, in particular protein sequences, which generally show >60% pairwise identity among sequences within each subgroup. Note that gene sequences containing tentative 5′-promoter and 3′-untranslated regions (UTR) have more rapid diversification (bottom left), indicating further divergence pertinent to epigenetic regulation than only genetic coding. IFN and IFNX, intron-containing and intronless type I IFNs; IFNL and IFNLX, intron-containing and intronless type III IFNs, respectively.

Direct examination of pairwise identity (%) from alignments of amphibian IFN protein sequences^38^, which are more conservative than their cDNA or genomic sequences, revealed 6–7 homologous subgroups: (1) XaIFN2-7, XaIFNX3, XaIFNX4, XaIFNX6, XaIFNX9, XaIFNLs-LXs; and three phylogenetic outliers (tentative progenitors, Fig. 2) of XaIFN1, XaIFNX11, and XaIFNX22, which have <50% identity to all others; and (2) XtIFN1-7, XtIFNX1, XtIFNX2, XtIFNX12, XtIFNX13, and XtIFNLs-LX. Each subgroup generally contains 2–12 relevant members with >50% pairwise residual identity, and most newly duplicated members may share 80–98% pairwise identity (Figs 5 and S1, upper right)^7^,^8^,^9^,^10^,^11^. Within each subgroup, gene sequences containing tentative 5′-promoter and 3′-untranslated regions (UTR) show much less similarity (Figs 5 and S1, bottom left), indicating further divergence pertinent to epigenetic regulation than only by genetic coding. As previously reported^9^, almost all frog IFNs conserve the four-cysteine residues, but show less sequence similarity in other residues/motifs between subgroups (Fig. S3a–d, Supplemental Excel Sheet 5). This explains why simple Blast searches have limited power to detect these distant IFN homologs, particularly for the intronless IFNs that have subthreshold sequence similarity to classic IFNs previously identified in frogs or other species^7^,^8^,^9^,^10^,^11^.

**Figure 6:**
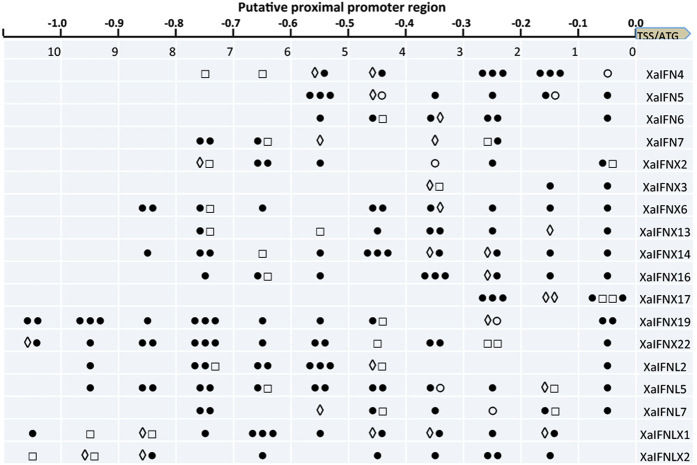
Categorization of IFN genes based on regulatory elements predicted in their proximal promoter regions. Regulatory elements (and pertinent binding factors) in the ~1 kb proximal promoter regions were examined against both human and animal TFD Database using a Nsite program (Version 5.2013, at http://www.softberry.com). Shown here are IFNs in *X. laevis,* whose promoters contain at least one IFN- or virus-stimulated response elements (ISRE, PRDI, and/or STAT1/3 factors). Other subgroups of IFN genes, whose promoters do not contain these IFN or virus regulatory elements, and a spreadsheet of all predicted regulatory elements (and relevant binding factors) are listed in the Supplemental Excel Sheet 6. *Legend*: ○, containing such as GATA-1 regulating constitutive expression; ◊, containing IFN-stimulated response element (ISRE) and PRDI to interact with IRF, ISGF3 and STAT factors; □, containing cis-elements interacting with factors to mediate immune/inflammatory responses including C/EBP, NF-kB, NF-IL6, and p53; ●, containing cis-elements reacting with other factors significant in other developmental/physiological responses.

**Figure 7:**
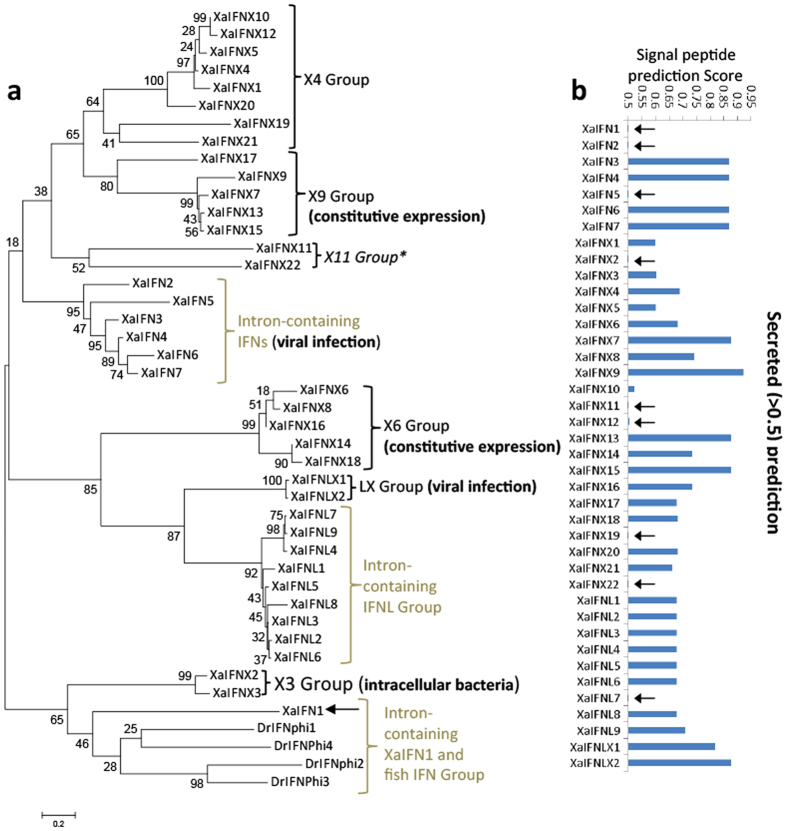
Molecular and functional subgroups of the IFN complex in *X. laevis*. (**a**) Evolutionary analyses were conducted in MEGA6, and the tree was inferred by using the Maximum Likelihood method based on the Poisson correction model^32^. Percentage of trees in which the associated taxa clustered together is shown next to the branches. In addition to the molecular subgroups based on their phylogenetic relationship at the molecular level, we inferred the functional subgroups (bold texts in parenthesis) based on overall examination of their gene and peptide sequences as well as experimental data. **(b**) Signal peptides of XaIFN precursors were examined using PrediSi (http://www.predisi.de) to determine the secretory potency of relevant IFN mature peptides, indicating the evolution of intracellular IFNs (indicated by arrows, signal peptide prediction score 0–0.5) in each subgroup, particularly of intronless IFNs. IFN and IFNX, intron-containing and intronless type I IFNs; IFNL and IFNLX, intron-containing and intronless type III IFNs, respectively.

We extensively analyzed cis-regulatory consensus and transcription factor-binding sites in putative proximal promoter regions within 1.5 kb upstream of the transcription start site (TSS) or start codon (ATG) of each amphibian IFN genomic sequence (Supplemental Excel Sheet 6 and 7)^39^. As expected, many regulatory elements (and their relevant binding factors) were detected within the IFN promoter regions. The first group we categorized was promoters containing IFN-stimulated response element (ISRE) and the positive-regulatory domain I (PRDI) involved in IFN- and virus-mediated induction. Both *cis*-elements (ISRE and PRDI) are in 18 of the 40 XaIFN genes and 14 of the 50 XtIFN promoter regions. The *cis*-elements of ISRE/PRDI, were identified in nearly one quarter (8 of 36) of XtIFNXs or half of the other IFN groups (i.e. IFNs, IFNXs, and IFNLs) in both amphibian species (Fig. 6, Fig. S4, and Supplement Excel Sheet 6 and 7)^40^. Notably, more than half of these IFN promoter regions do not contain IFN- or virus-responsive elements, but have regulatory elements interacting with factors to mediate immune/inflammatory responses including C/EFB, NF-kB, NF-IL6, and p53. This suggests an inductive expression response to other immune or inflammatory signaling, such as those mediated by Toll-like receptors (TLRs)^41^. Several of these IFN promoters contain elements such as GATA-1, which often promote constitutive expression^42^. This *in silico* promoter analyses categorized the amphibian IFN complex generally into three subgroups: (1) IFN- and virus-responsive, (2) immune- and inflammation-signaling mediated by highly conserved pathogen/danger recognition molecules, and (3) constitutively expressed genes that are likely relevant to normal developmental/physiological processes (Fig. 7a)^40^,^41^,^42^. IFNs are generally thought to initiate antiviral responses through secreted mature peptides that bind the ectodomains of membrane-bound receptors^1^,^2^,^3^,^4^,^5^. Recent studies shed light on potential intracellular signaling by some fish IFNs^25^. Our studies of amphibian IFNs also revealed that multiple amphibian IFNs contain no signal peptides for secretion. These IFNs include XaIFN1, XaIFN2, XaIFNX2, XaIFNX11, XaIFNX12, XaIFNX19, XaIFNL7 in *X. laevis*, and many XtIFNs in *X. tropicali* too (Fig. 7b, Fig. S2, and Supplemental Excel Sheet 8). Further study of the likely intracellular signaling mechanisms of these amphibian IFN peptides will lead to a more comprehensive understanding of IFN signaling^25^,^43^.

### Sub-group determination of constitutive and induced expression, and cloning of representative IFNs

**Figure 8:**
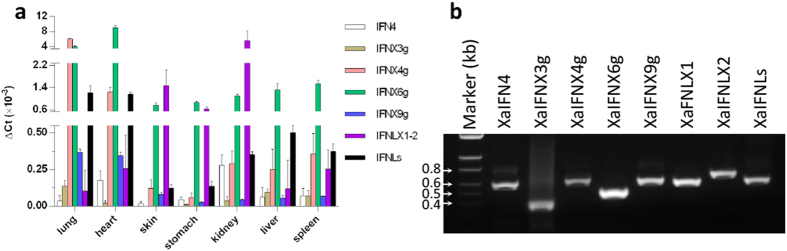
Family-wide expression analysis of *X. laevis* IFN genes (IFNs) in different tissues. (**a**) Gene expression was analyzed using a SYBR Green-based real-time RT-PCR assay. Total RNA (100 ng) was used in each 20 μl of PCR reaction. Ct values of genes were normalized against Ct values of a housekeeping gene (beta-actin) amplified from the same RNA samples to obtain 2^−ΔCt^, reflecting expression of each subgroup of IFN genes relative to beta-actin. Data are means ± SE; n = 3 replicates. (**b**) Representative IFN cDNA was amplified from a pooled RNA sample for cloning purposes. Primers for RT-PCR detection and cloning are listed in Supplemental Excel Sheet 9.

We have performed experimental studies of the amphibian IFN complex primarily using the *X. laevis* system. Subtype-common or isoform-specific primers intended for expression analyses using quantitative RT-PCR and cloning of coding regions were generated based on sequence alignments (Supplemental Excel Sheet 9)^10^. Expression of different subgroups of IFN transcripts was determined in various tissues from adult African clawed frogs (gift from Dr. Peying Fong, Department of Anatomy and Physiology, Kansas State University). Compared with the expression of the β-actin internal control, constitutive expression of the intronless XaIFNX6 group (IFNX6g, including XaIFNX6, X8, X14, X16, and X18, see Fig. 8a) was detected in all tested tissues with especially strong expression in lung and heart. Similarly, the XaIFNX4 group (IFNX4g, including eight intronless XaIFNs), the XaIFNX9 group (IFNX9g, including XaIFNX7, X9, X13, and X15), and the XaIFNL group (IFNLs, including XaIFNL1-9) were strongly expressed in lung and heart, and also were expressed in kidney, liver, and spleen. The two intronless type III IFNs (XaIFNLX1-2) were expressed in skin, stomach, and kidney. The intronless XaIFNX3 group (IFNX3g, containing XaIFNX2 and X3) had the lowest tissue expression but was detectable in lung, liver and spleen. The intron-containing type I IFN4 had the highest expression in kidney and heart (Fig. 8a). Expression of intron-containing type III IFNs has been described in the *X. tropicalis*^9^, but the existence and expression of intron-containing and intronless type I IFNs have not been studied. Through analysis of RNA-Seq data associated with the current genome assembly of *X. tropicalis* (*Silurana*) at NCBI, qualified RNA reads were mapped within the transcripts of most intronless XtIFNXs (XtIFNX1-24 and XtIFNLX1) (Fig. S5), even though the genes of these amphibian IFNXs have not been annotated. In addition to amplification of their short fragments using PCR detection, we also successfully extracted the coding regions from pooled cDNA for all subgroups using the cloning primers covering whole open reading frames (ORF) (Fig. 8b), and identified representatives of each subgroup through the cloning process.

**Figure 9:**
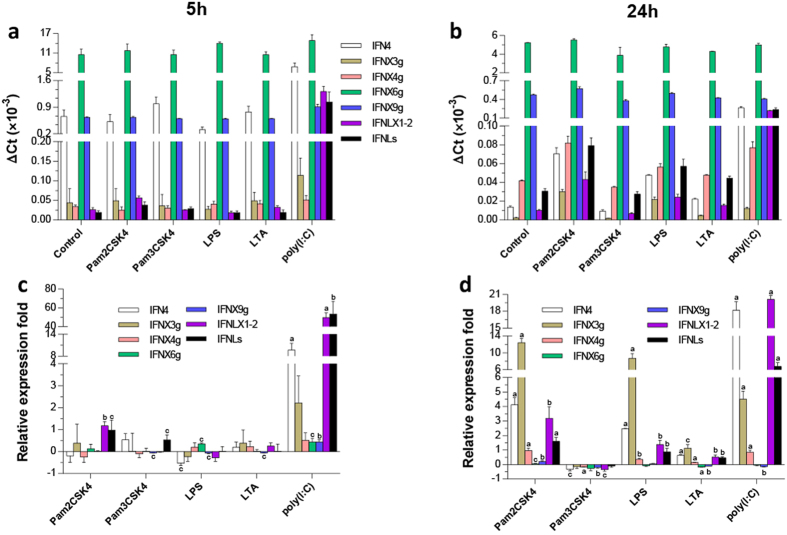
Induced expression analysis of *X. laevis* IFN genes (IFNs) in a kidney-derived cell line (A6) treated with pathogenic mimics for 5 (**a**,**c**) or 24 h (**b**,**d**). Gene expression was analyzed using a SYBR Green-based real-time RT-PCR assay. Total RNA (100 ng) was used in each 20 μl of PCR reaction. Ct values of the genes were normalized against Ct values of a housekeeping gene (beta-actin) amplified from the same RNA samples to obtain 2^−ΔCt^ (**a**,**b**), which reflect the expression of each subgroup of IFN genes relative to beta-actin; or to normalize for fold changes to control (mock) stimulation (**c**,**d**). Data are means ± SE; n = 3 replicates of 2–3 independent assays, *(**a–c**) *p* < 0.001, 0.01, and 0.05, respectively, compared to control. Pam2/3CSK4, LTA and LPS are bacterial mimics to act through TLR2/6, TLR2/1, TLR2, and TLR4 pathways, respectively; and poly (I:C) is a mimic of viral dsRNA. Primers for RT-PCR detection and cloning are listed in Supplemental Excel Sheet 9.

Using a cell line derived from *X. laevis* kidney tissues (A6 CCL-102™ ATCC^®^, Manassas, VA), we further studied the constitutive and induced expression after cells were stimulated with different microbial mimics. In Fig. 9, we plotted these expression data in two ways: the upper panel was standardized to the internal house-keeping gene β-actin to show constitutive expression (Fig. 9a,b), and the bottom panel was normalized to the control treatment for induced expression from microbial stimuli (Fig. 9c,d). Constitutive expression (Fig. 9a,b) of the intronless XaIFNX6 group and XaFINX9 group was consistently strong in control and treated cells, particularly in the early phase (5 h) of the treatments. Intron-containing IFNs (represented by IFN4) was constitutively expressed at 5 h, but a more inductive pattern became obvious after cells were treated for 24 h. The viral dsRNA mimic, poly (I:C), was most effective for stimulation of several subgroups of XaIFNs, including intron-containing IFN4, intronless IFNX3g, and two subgroups of type III IFNs (IFNLX1-2 and IFNLs) at either 5 h or 24 h post treatment (Fig. 9c,d). TLR2 and TLR4 ligands (Pam2/3CSK4/LTA, and LPS, respectively) stimulated the expression of these same IFN subgroups especially at 24 h post treatment. Significant upregulation was observed in treatments with the ligand for TLR2/6 (Pam2CSK4) and TLR4 (LPS), but not ligands for TLR2/1 (Pam3CSK4) or TLR2 only (LTA), indicating differential responses among amphibian IFN subgroups pertinent to innate immune responses against different pathogens^40^,^41^,^44^. Collectively, the expression of these amphibian IFNs, in particular the novel intronless IFNs, is experimentally evident *ex vivo* and differentially expressed in cells indicating functional diversity as components of the innate immune system of amphibians.

### Involvement of amphibian IFNs in cellular responses to infections by influenza virus

Influenza A viruses (IAVs) infect multiple animal species globally and threaten both animal and human health^45^,^46^. Horizontal transmission of IAVs between wild and domestic animals especially waterfowl, chickens, and pigs results in annual influenza outbreaks. Reptiles and amphibians are considered as potential vectors/hosts harboring IAVs, but their role in IAV cross-species transmission has received little attention^45^,^46^. To examine this potential epidemiological link, we tested the susceptibility of frog cells to different subtypes of IAVs isolated from several animal species, including avian H9N2, equine H3N8, human H1N1 and swine H1N2 and H3N2 viruses. Compared with the IAV strains isolated from chickens and horses, the pig and humans isolates showed higher infectivity in frog cells (Fig. S7a), indicating that IAVs of different animal origins likely differ in specificity to bind cellular receptors leading to distinct infectivity in frog cells. In particular, an H3N2 porcine TX98 isolate, demonstrated a significant degree of infectivity in frog cells, indicating a potential, well-adapted compatibility of virus-host interaction. Pigs serve as a “mixing vessel” for different IAVs, resulting in novel strains causing zoonotic infection of animals and humans^45^. Our data suggests that amphibians, which are in close contact with and are frequently prey for waterfowl and wild pigs in watering habitats, could be an overlooked intermediate hosts for IAV evolution and transmission^47^.

**Figure 10:**
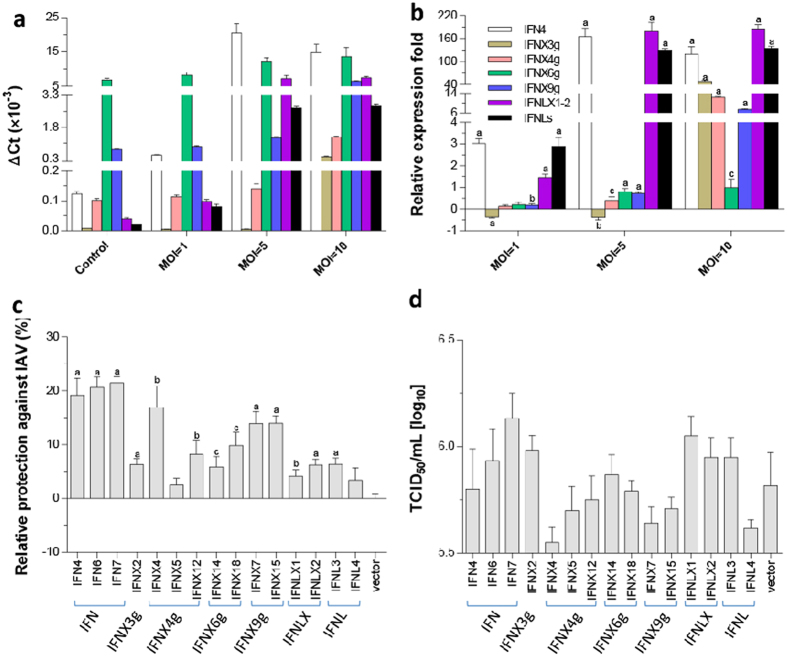
Induced expression and antiviral activity of *X. laevis* IFN genes (IFNs) in amphibian kidney A6 cells. (**a**,**b**) Cells were infected for 48 h by a swine influenza virus (TX98 strain) at the MOI of 1, 5, or 10. Gene expression was analyzed using a SYBR Green-based real-time RT-PCR assay as previously defined. Ct values of the genes were normalized against Ct values of a housekeeping gene (β-actin) amplified from the same RNA samples to obtain −ΔCt (**a**), which reflect the expression of each subgroup of IFN genes relative to beta-actin; or 2^−ΔCt^ normalized to get fold changes relative to the non-infected control (**b**). (**c**) Sequence-confirmed overexpression constructs (in a pcDNA3.3 Topo-mammalian expression vector, Invitrogen) representing each group of *X. laevis* IFNs were transfected into A6 cells (0.25 μg DNA/well in 96-well culture plates, >60% transfection efficacy). The cells were then infected with the TX98 virus at 5 MOI. The protection of IFNs from the viral infections was then quantified at 2 days post infection using a crystal violet staining procedure. (**d**) Virus titers in the cells treated with different amphibian IFNs as in (**c**). The cells were transfected with different amphibian IFNs as described in the (**c**), then infected with the TX98 virus at a MOI of 5. Culture supernatants were collected for virus titration using an endpoint dilution assay to define 50% tissue culture infective dose (TCID50) in MDCK cells. Data represent results of two independent experiments. Data are means ± SE; n = 3 replicates, (**a–c**) *p* < 0.001, 0.01 and 0.05, respectively, compared to control. Primers for RT-PCR detection and cloning are listed in Supplemental Excel Sheet 9.

Infection of frog kidney cells by porcine TX98 strain, stimulated expression of intron-containing IFN4 and type III IFNs, including two intronless IFNLX and classic intron-containing IFNLs. A dramatic more than 100-fold increase of these virus-responsive IFNs was observed in cells infected with the virus at an MOI of 5 or 10 for 48 h. In addition, robust upregulation of most intronless type I IFNs was also detected in cells infected with the virus at 10 MOI, indicating a strong IFN response upon a massive viral infection (Fig. 10a,b). Antiviral assays by direct transfection of A6 cells using IFN-expressing constructs, indicated that most of the 15 tested amphibian IFN subtypes, which comprise 1–3 typical candidates of each subgroup, exerted significant antiviral protection in A6 cells. Only IFNX5 of the IFNX4g group and two constructs (IFNX14 and IFNX18) of the IFNX6g group were less protective of cells from virus infection. These same two groups were also less responsive to IAV-induced IFN expression (Fig. 10c). Viral titers were also determined from virus-containing supernatant collections of different IFN treatments. In general, the tested intron-containing IFN group (including IFN4, IFN6, and IFN7), which had higher activity to protect cells from virus-induced cytopathic effect, exert lower activity in suppression of viral replication. In contrast, the IFNs of intronless groups conferred higher virus-suppression but were less effective in protecting cells from cytopathic effect. Type III IFNs tested mostly had less potency in either cell protection or viral suppression; however, IFNL4 showed strong virus-suppressive activity than most type I IFNs tested (Fig. 10c,d). Throughout the tests, IFNX4 was remarkable for its strong virus-suppressive activity as well as cell protective activity similar to intron-containing IFNs (Fig. 10c,d). Addition of overexpressed IFN peptides into culture medium of A6 cells also stimulated protection against IAV-induced cytopathic effect similar as direct transfection (Fig. S8). Clearly, our *in vitro* antiviral findings indicate that the IFN complex of amphibians provides profound antiviral diversity regarding their antiviral potency against IAV infection, but calls for further characterization *in vivo*.

### Diverse activity against infections by the intracellular bacterium, *L. monocytogenes*

**Figure 11:**
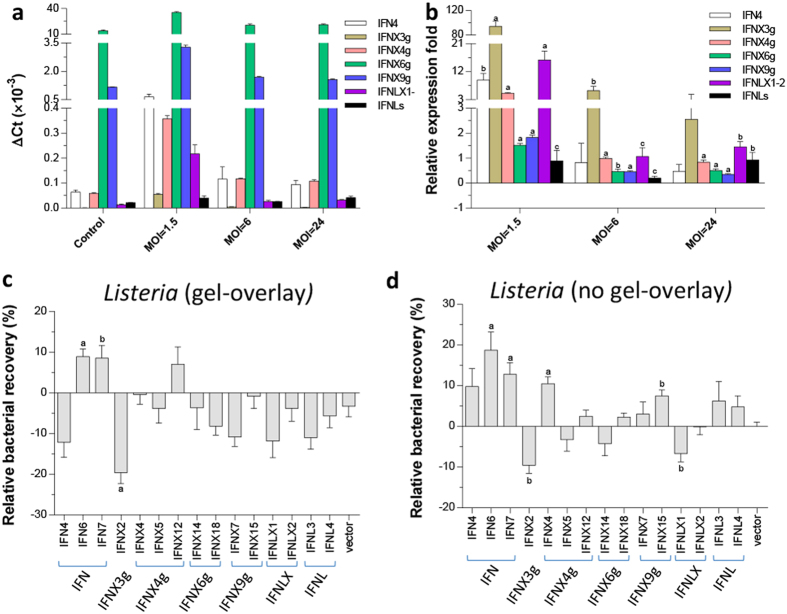
Induced expression and antibacterial activity of *X. laevis* IFN genes (IFNs) in amphibian kidney cells (A6). (**a**,**b**) Cells were infected by an intracellular bacterium, *L. monocytogenes* (ATCC 19115) at 1.5, 6 and 24 MOI. Bacterial infection was performed using an agarose-gel-overlay procedure to limit intracellular infection during the test periods. Gene expression was analyzed using a SYBR Green-based real-time RT-PCR assay as described above. (**c**,**d**) Sequence-confirmed overexpression constructs (in a pcDNA3.3 Topo-mammalian expression vector, Invitrogen) representing each groups of *X. laevis* IFNs were transfected into A6 cells (1.0 μg DNA/well in 24-well cell culture plates, >60% transfection efficacy). Cells were then infected with the bacteria at 1.5 MOI for 1 h, washed and overlaid with the medium containing 0.7% agarose and gentamicin (10 μg/ml) (**c**), or cultured without gel overlay but with the medium containing gentamicin (**d**). Bacterial loads from the cell culture were then quantified at 72 h post infection using an alamarBlue bacterial quantification procedure (AbD Serotec). Data are means ± SE; n = 3 replicates, **(a–c**) *p* < 0.001, 0.01 and 0.05, respectively, compared to control. Primers for RT-PCR detection and cloning are listed in Supplemental Excel Sheet 9.

Interferons provide broad protective immunity against other intracellular pathogens in addition to cell-obligate viruses^1^,^2^. To determine if amphibian IFNs also mediate immune responses against other intracellular pathogens, we established an infection in A6 cells using a prevalent intracellular bacterial pathogen, *L. monocytogenes*. Unlike viruses, *L. monocytogenes* has a saprophytic lifestyle not solely dependent on intracellular growth^48^. To confine it to an intracellular lifecycle, we used a gel-overlay procedure^48^. Most amphibian IFNs were significantly upregulated when cells were infected intracellularly with bacteria at a low 1.5 MOI for 24 h. Throughout all infection tests with different bacterial dosages, expression of the intronless IFNX3 group was exceptionally high, indicating that this intracellular IFN subtype (see Fig. 7) may have differentiated in order to stimulate antibacterial activity inside cells (Fig. 11A,B). In cell cultures with a gel-overlay, A6 cells transfected with most IFN-expressing constructs were antibacterial to some extent, except for IFN6, IFN7, and IFNX12 (Fig. 11c). However, most IFNs were much less active without a gel-overlay, which limits the bacterial growth intracellularly (Fig. 11d). Surprisingly, IFNX2, the intracellular IFN isoform, exerted significant antibacterial activity in both test conditions (Fig. 11c,d). Correspondingly, the most antibacterial effective IFNX3 group contains intronless IFNX2 and IFNX3, which were most responsive in cells infected with *L. monocytogenes* (Fig. 10a,b), or treated with ligands for either TLR2 (Pam2CSK4) or TLR4 (LPS) (Fig. 9b,d). Considering that IFNX2 represents an intracellular IFN peptide that lacks a signal peptide for secretion (Fig. 7), it is likely that IFNX2 (probably other antibacterial effective IFNs as well) induces protective activity against intracellular bacteria via intracellular signaling pathways different from classical IFN signaling process^25^,^43^,^48^. In summary, we have demonstrated that the amphibian IFN family confers differential expression patterns and exerts diverse antiviral and antibacterial activity. Furthermore, some IFN subtypes have evolved to provide protection against infections by transboundary pathogens such as influenza virus and environmental pathogens such as *L. monocytogenes*.

### Induction of IFN-stimulated genes (ISGs)

**Figure 12:**
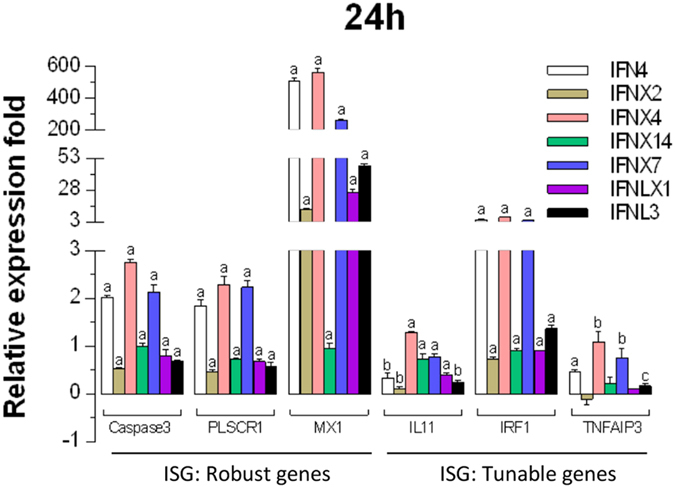
Amphibian IFNs induced expression of IFN-stimulated genes (ISGs) in frog cells. Frog cells at 80% confluence were treated with overexpressed IFN peptides (20 ng/ml) for 24 h. Gene expression was analyzed using a SYBR Green-based real-time RT-PCR assay. Total RNA (100 ng) was used in each 20 μl of PCR reaction. Ct values of the genes were normalized against Ct values of a housekeeping gene (beta-actin) amplified from the same RNA samples to obtain 2^−ΔCt^, which reflects the expression of each subgroup of IFN genes relative to beta-actin and were further normalized for fold changes to the control (mock). Data are means ± SE; n = 3 replicates of 2–3 independent assays, *(**a–c**) *p* < 0.001, 0.01, and 0.05, respectively, compared to control. Abbreviations: IL11, interleukin 11; IRF1, IFN-regulatory factor 1; MX1, an IFN-induced dynamin-like GTPase; PLSCR1, phospholipid scramblase 1; and TNFAIP3, tumor necrosis factor alpha-induced protein 3. Primers for RT-PCR detection and cloning are listed in Supplemental Excel Sheet 9.

Innate immune IFNs exert antiviral and immunomodulatory activity through induction of hundreds IFN stimulated genes (ISGs), which are generally classified into robust or tunable ISGs relative to their responsive intensity to IFN stimulation^1^,^2^,^3^,^4^,^5^,^6^,^14^. Whereas most robust ISGs are involved in antiviral responses, tunable ISGs are more broadly modulatory for immune and developmental regulation^1^,^6^,^14^. To test the potency of these newly identified amphibian IFNs in induction of ISGs, we measured the expression of six typical ISGs (three robust and three tunable)^6^ in frog cells treated with the overexpressed IFN peptides. Data show that IFN peptides from each functional group significantly stimulate all three robust ISGs including caspase 3, phospholipid scramblase 1 (PLSCR1), and an IFN-induced dynamin-like GTPase (MX1). Amphibian IFN4, IFNX4 and IFNX7 stimulated MX1 200–500-fold higher than their expression levels in control cells at 24 h post treatment. Amphibian IFN peptides also upregulated the expression of three tunable ISGs, including interleukin 11 (IL11), IFN-regulatory factor 1 (IRF1), and tumor necrosis factor alpha-induced protein 3 (TNFAIP3); they were generally significantly induced but to a lesser extent at 1–3-fold increase as compared with those in control cells (Fig. 12). In summary, amphibian IFNs including these newly identified intronless IFNs, are capable of induction of a serial ISGs to mount diverse antimicrobial activity^1^,^2^,^3^,^4^,^5^,^6^,^14^,^15^.

### Revised model of IFN evolution and immunobiology

**Figure 13:**
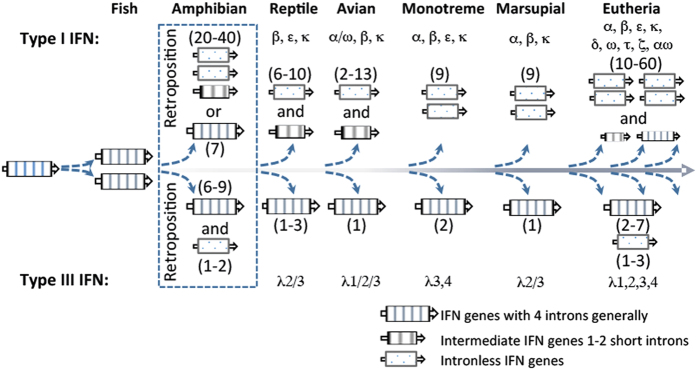
Revised model for the evolution of type I and type III IFNs in vertebrates. This model shows intronless type I IFNs originating and expanding in amphibians with independent diversification in amniotes thereafter. In contrast, the classical IFN evolution model (Fig. 1), depicted a linear increase of IFN complexity from fish to eutherian animals. Discovery of IFN expansion in amphibians highlights two large IFN expansions in both amphibian and eutherian species, which then envisions a non-linear IFN evolution process throughout jawed vertebrates. Conversely, type III IFNs are preeminent for their conserved intron-containing gene structures and family numbers throughout vertebrates. One to several intronless type III IFNs were also identified in amphibians, as well as in many eutherian species. Given the similar antiviral signaling evoked by type I and type III IFNs, it remains unknown why intronless type III IFNs underwent little expansion and even elimination in contrast to the intronless type I IFN genes in most vertebrate species.

Our identification of the coexistence and expansion of intronless IFNs in frogs revises the previous evolution model of antiviral IFNs in vertebrates in several aspects (Fig. 13 versus Fig. 1)^7^,^8^,^9^,^10^,^11^. Significant conclusions of these studies were: (1) the emergence of intronless IFNs occurred in amphibians before reptiles^8^,^9^; (2) the coexistence of intron-containing IFN progenitors and expansion of intronless IFNs emphasizes the uniqueness of amphibians in evolutionary immunology^44^; and (3) the most complicated IFN system (in both molecular types and total numbers of IFNs compared with other species) evolved in amphibians. This seems a surprise but should not be unexpected, which may partly reflect the most dramatic lifestyle adaptation during vertebrate evolution, i.e., leaving water to adapt to terrestrial environments. Interestingly, amphibian intronless IFNs have less similarity than their intron-containing IFNs to the intronless type I IFNs in birds or mammals, indicating that the emergence and expansion of intronless IFNs in amphibians may not be an evolutionary continuum (but an independent bifurcation) leading to intronless IFNs that dominate in amniotes^7^,^8^,^9^,^10^,^11^. It is likely that they shared the same origin but differed independently thereafter in frogs and other amniote species. Previously, type III IFNs identified in all vertebrate species primarily conserved their intron-containing gene structures^4^,^5^. As a continuation of identification of intronless type III IFNs in amphibians, we have inspected available genomes of most tetrapod species and found that the existence of intronless type III IFNs is not unique to frogs, and at least one gene of intronless IFN-λ1 has been identified in many eutherian species. Most interestingly, intronless IFNLs appear to have expanded in different species of bats, which generally harbor 2–3 genes of intronless IFN-λ2/3 (data not shown), thus serving as a good molecular model to study host-pathogen coevolution leading to expansion of type III IFNs. As predicted from this model, we expect to identify the coexistence of both intronless and intron-containing IFNs in other amphibian species as well as potentially from some species of lungfish, given that their genome sequences will be available soon. In summary, our discoveries of the coexistence and expansion of intronless IFNs in amphibians revise the time/species theory of IFN genetic evolution as previously stated to theoretically explain the occurrence of intronless IFNs in jawed vertebrates. Revising this theory towards initials evolution in amphibians provides insight and a basis for reexamining the current evolutionary model of IFN cytokine development^7^,^8^,^9^,^10^,^11^. This may lead to cross-species identification of novel groups of IFNs that provide protection against transboundary or zoonotic infections (Fig. 12).

Functionally, type I and III IFNs have been primarily studied for their roles in antiviral regulation; however, recent studies imply their multifunctional propensity in regulation of other immune and physiological process^1^,^2^,^3^,^4^,^5^,^12^,^13^,^14^,^15^,^16^,^17^,^18^,^19^,^20^,^21^,^22^,^23^. The identification of the most complicated IFN complex in amphibians furthers this postulation. First, many amphibian IFNs have a constitutive expression pattern likely corresponding to developmental and physiological regulation rather than induced responses to infections^12^,^13^,^14^,^15^,^16^,^17^,^18^,^19^,^20^,^21^,^22^,^23^. Second, the differential expression patterns and cellular locations demonstrate temporal and spatial diversity among IFN subgroups/isoforms in an animal species. Finally, the coexistence of significant members of both intron-containing and intronless IFNs not only highlights the molecular transition but also the required functional coordination, which therefore envisions cross-subtype optimization of IFN signaling for both antiviral and homeostatic regulation^12^,^13^,^14^,^15^,^16^,^17^,^18^,^19^,^20^,^21^,^22^,^23^. Amphibians, such as frogs and toads, might carry zoonotic pathogens such as influenza viruses and *Listeria;* however, their roles as vector/reservoir organisms have not been well studied compared with waterfowl and pigs^45^,^46^. Swine influenza viruses exhibited significant infectivity in frog cells with much higher compatibility than IAV strains isolated from birds, horses, and humans. Although domestic pigs have limited contact with amphibians, a wild pig wallowing in shared water habitats with amphibians could consume more than 1,000 reptiles and amphibians annually^47^. Thus, if pigs serve as mixing vessels for co-evolution of influenza viruses^45^, amphibians may contribute to influenza diversity. Amphibians as typical insect eaters could also be critical vectors for other arthropod-borne viruses (arboviruses) and intracellular bacteria, which are a major health threats in both humans and animals^46^,^49^. Coordinated regulation of protective responses mediated by IFN signaling in both vector animals and end hosts may provide an integrated approach to discern critical components of conserved, cross-species innate immune mechanisms. These future studies may in turn provide means to control vectored and/or zoonotic infections^44^,^50^.

## Methods and Materials

### Ethics statement, animal tissues, and cells

No living animals were involved in this study; the frog carcasses used for dissection and tissue collection were gifts from an electrophysiological laboratory (Dr. Peying Fong, at the Department of Anatomy and Physiology, Kansas State University), where African clawed frogs (*X. laevis*) are routinely used. The Kansas State University Biosafety and Institutional Animal Care and Use committees approved all recombinant DNA procedures and animal procedures. Samples of various tissues were collected, immediately snap frozen and stored in liquid N_2_ until used for nucleic acid extraction. The A6 cell line, derived from *X. laevis* kidney, was purchased from American Tissue Culture Collection (CCL-102™ ATCC^®^, Manassas, VA). Ligand components, including Pam2/3CSK4, LPS, LTA, and poly (I:C) used for stimulation of cells, were purchased from InvivoGen (San Diego, California) and applied to cell cultures at 0.1–10 μg/ml for 5 or 24 h as indicated.

### Infectious agents

All experiments using bacteria and viruses were conducted in the laboratories covered by effective licenses and handled according to restrictive regulations specified. The bacterium used was *Listeria monocytogenes* (ATCC 19115) and transferred from ATCC. Viruses tested include human H1N1 A/California/04/2009 (CA09), porcine H3N2 A/swine/Texas/4199-2/98 (TX/98) and variant H1N2 A/swine/Kansas/12-156064/2012 (vH1N2), avian A/quail/Hong Kong/G1/1997 (H9N2), and equine A/equine/Kansas/E1/2011 (H3N8) strains. Procedures for viral and bacterial infections were conducted in cells as previously described^50^,^51^. Cytopathic effect (CPE) and immunochemical staining of viruses were used to measure viral infectivity and titers, and a plaque assay and AlamarBlue kit (AbD Serotec, Raleigh, NC) were used to measure IFN-mediated antibacterial activity in A6 cells^48^,^51^.

### Bioinformatics and phylogenetic procedures for sequence analyses

A combinative procedure was used to identify the existence of intronless IFN genes in amphibian genomes^10^. Single and grouped sequences, or hidden Markov model (HMM) profiles generated using sequence alignments with identified IFN peptide sequences in fish, birds, mammals, and amphibians, were used to query of three genome assemblies: *X. tropicalis* (*Silurana*) at NCBI (http://www.ncbi.nlm.nih.gov/genome/80, submitted by the DOE Joint Genome Institute), *X. tropicalis* 9.0 genome (*Nigerian*) and *X. laevis* J-strain 9.1 at Xenbase (http://www.xenbase.org/). The protein BLAST searches were conducted using the default algorithm parameters with BLOSUM62 matrix and Expected thresholds (E) less than 10 or 1. Resultant amphibian IFN homologs (including most intron-containing IFNs and >20 intronless IFNs) were further used as query entries to inspect other more diversified IFN homologs, which appears to have very little pairwise identity (<40%) to intron-containing frog IFNs previously identified.

The IFN genes were predicted and extracted from genomic sequences, which span the regions having translated frames significantly similar (~50% peptide similarity and E < 10^−5^) to identified IFN sequences or consensuses. Repetitive elements including retrotransposons within the interested genomic sequences were determined using the RepeatMasker program (version 4.0.6, http://www.repeatmasker.org/). Programs interactively used for gene prediction include GenomeScan (http://genes.mit.edu/genomescan.html) and FGENESH (http://www.softberry.com), and were further manually annotated for confirmation. Peptide sequences were translated using the translate tool at the ExPASy port, and signal peptides were examined using PrediSi (http://www.predisi.de). Sequence alignments were generated primarily using the programs of MUSCLE and ClustalW through EMBL-EBI port (http://www.ebi.ac.uk/), and other sequence management was conducted using programs at the Sequence Manipulation Suite (http://www.bioinformatics.org). Regulatory elements in the putative proximal promoters were examined against both human/animal TFD Database using program Nsite (Version 5.2013, at http://www.softberry.com)^39^. Visualization of sequence alignments was conducted using Jalview, phylogenetic analyses using MEGA6^32^, recombination analyses using SDT and RDP4^52^, and topological comparison between the Newick trees was performed with Compare2Trees (http://www.mas.ncl.ac.uk/~ntmwn/compare2trees). Other than indicated, all programs were run with default parameters^10^.

### Gene identification, expression analyses, and cloning

Based on sequence analyses, we designed subtype-common or gene-specific primers for expression analyses using quantitative RT-PCR and cloning of coding regions from cDNA pools (Supplemental Excel Sheet 9)^10^. For validation of the expression of various subgroups of frog IFNs, we amplified cDNA covering whole coding ORFs of representative genes in each subgroup, cloned them in a pcDNA3.3 Topo-mammalian expression vector (Invitrogen, Carlsbad, CA), and confirmed them by sequencing. The cDNA was reverse transcribed from total RNA pools extracted from different tissues and poly (I:C) stimulated A6 cells with a SuperScript III first-strand synthesis system and random primers (Invitrogen). Coding regions of IFNs were amplified from this cDNA pool for transcription confirmation and building expression constructs. PCR optimization, and real-time RT-PCR analysis were performed as described^10^. In brief, gene-specific or subtype-common primers were designed based on multiple alignments of related IFN sequences, and PCR conditions were optimized and validated using confirmed IFN plasmids to show specific amplification only with templates containing confirmed IFN clone(s). RNA was extracted from tissues and cells as described above. Real-time RT-PCR assays were conducted in a 96-well microplate format using a StepOnePlus™ Real-Time PCR System (Applied Biosystems, Grand Island, NY) with the validated primers. Reactions were conducted with a SYBR Green RT-PCR system (Qiagen, Valencia, CA) with 100 ng of total RNA in a 20-μl reaction mixture. Specific optic detection was set at 78 °C for 15 s after each amplification cycle of 95 °C for 15 s, 56–59 °C for 30 s and 72 °C for 40 s. Critical threshold (Ct) values and melt curves were monitored and collected with an enclosed software. Relative gene expression was first normalized against Ct values of the housekeeping gene (β-actin) for relative expression levels, and compared with the expression levels of control samples for stimulated regulation^10^.

### Functional analysis against transboundary pathogens

Three to five clones of each IFN subgroup were selected by PCR and sequencing confirmation. Two or three clones with the highest sequence identity (98–100%) to the template sequence extracted from Xenbase genomic sequences were used for functional studies. Endotoxin-free plasmids (equal to 0.25 μg/well in 96-well cell culture plates) from confirmed clones were purified from individual bacteria cultures and used for cell transfections in A6 frog cells for transient expression and antimicrobial assays^10^,^48^,^51^,^53^. For peptide production, IFN-expression plasmids were transfected in HEK293F cells of a mammalian-expression system (Invitrogen) to produce IFN peptides as previously described^10^. Antimicrobial assays were conducted as described^10^,^48^,^51^,^53^. Induction of IFN-stimulating genes (ISGs), exemplified for both robust and turntable ISGs, was determined in cells treated with indicated amphibian IFN peptides (20 ng/ml) in 24-well cell culture plates^10^,^48^,^51^,^53^.

### Nucleotide sequence accession numbers

Nucleotide sequences for the new identifications, characterizations, and sequences in this article have been deposited in GenBank, with consecutive Accession numbers between KU594511 - KU594560 and KU594561 - KU594600 for IFN entries from *X. tropicalis* and *X. laevis*, respectively (Table S1, and Supplemental Excel Sheet 1).

## Additional Information

**How to cite this article**: Sang, Y. *et al*. Expansion of amphibian intronless interferons revises the paradigm for interferon evolution and functional diversity. *Sci. Rep.* **6**, 29072; doi: 10.1038/srep29072 (2016).

## Supplementary Material

Supplementary Information

Supplement Excel Sheet

## Figures and Tables

**Table 1:** Enrichment and association of retrotransposons with phylogentically more primitive intronless IFNs in *X. laevis*.

**IFN gene**	**Associated Retrotransposon (RT)**	**% of RT within IFN gene-centered region**	**Phylogenic relevance (%)**	
XaIFN1	3 3′-LTR/Gypsy	1.9 (Chr9_10S)	N/A	
XaIFN3	1 5′-LTR/Gypsy			
XaIFN7	2 3′-LTR/Gypsy			
XaIFNX1	2 3′-NonLTR/Tx1;			
1 3′-LTR/Gypsy		5.7 (Chr9_10L)	60.5%	
XaIFNX5	2 3′-NonLTR/Penelope			
XaIFNX6	1 3′-LTR/DIRS			
XaIFNX9	1′ 5′-NonLTR/L1			
XaIFNX10	1′ 3′-NonLTR/L1			
XaIFNX11	1′ 3′-LTR/Gypsy			
XaIFNX18	2 5′-NonLTR/CR1			
**XaIFNX19**	**2 3**′**-LTR/DIRS; 5 3**′**-LTR/Gypsy**	15 (Chr3L)	76.2%	
**XaIFNX20**	**1 5**′**-LTR/Gypsy**			
**XaIFNX21**	**1 3**′**-LTR/Gypsy**			
**XaIFNX22**	**2 5**′**-LTR/Gypsy; 4 3**′**-LTR/Gypsy**			
**XaIFNLX1**	**4 3**′**-LTR/DIRS**	9.4 (Chr3L)	N/A	
XaIFNLX2	n/a			
XaIFNL1	1 3′-NonLTR/L2	5.2 (Chr8S)	70.0%	
XaIFNL2	1 3′-LTR/Gypsy			
XaIFNL3	1 3′-LTR/Gypsy			
XaIFNL4	1 3′-LTR/Gypsy			
XaIFNL5	1 5′-LTR/Gypsy			
XaIFNL6	1 3′-NonLTR/Penelope			
XaIFNL8	2 5′-NonLTR/Penelope; 1 5′-LTR/Gypsy			
