# Supplementary material for: Expansion of amphibian intronless interferons revises the paradigm for interferon evolution and functional diversity
Source: Sci Rep. 2016 Jun 30;6:29072. doi: 10.1038/srep29072 (PMC4928184; doi:10.1038/srep29072)
Supplement: Supplementary Information [file srep29072-s1.pdf]

Title: Expansion of amphibian intronless interferons revises the paradigm for interferon evolution and functional diversity

Yongming Sang, Qinfang Liu, Jinhwa Lee, Wenjun Ma, D. Scott McVey, Frank Blecha

Table S1: Genomic location and predicted cDNA and peptide length of amphibian IFN complex.

Amphibian IFNs	GenBank Acc#	Genome loc. (NCBI)	Genome loc. (Xenbase)	ORF (bp)	Peptide (AA)	Note
X. tropicalis			***Chr:10 (Assembly 9.0)***			
XtIFN1	KU594511		29491919-29488526	567	188	
XtIFN2	KU594512		29476492-29472563	639	212	
XtIFN3	KU594513		29463159-29460658	639	212	
XtIFN4	KU594514		29450672-29448761	570	189	
XtIFN5	KU594515		29439201-29435864	585	194	
XtIFN6	KU594516		29454973-29453021	570	189	
XtIFN7	KU594517		29444204-29442281	561	186	
		Scaffold NW_004668255.1	***Chr:03***			
XtIFNX1	KU594518	779099-778518	129027704-129028285	582	193	
XtIFNX2	KU594519	781375-780803	129029989-129030561	573	190	
XtIFNX3	KU594520	783281-782487	129031673-129032467	795	264	
XtIFNX4	KU594521	786516-785800	129034986-129035576	717	238	
XtIFNX5	KU594522	789617-788901	129038087-129038677	717	238	
XtIFNX6	KU594523	791988-791407	129040593-129041174	582	193	
XtIFNX7	KU594524	794073-793501	129042687-129043142	573	190	
XtIFNX8	KU594525	795936-795355	129044541-129045122	582	193	
XtIFNX9	KU594526	799459-798743	129047929-129048519	717	238	
XtIFNX10	KU594527	802191-801610	129050796-129051251	582	193	
XtIFNX11	KU594528	804533-803952	129053138-129053710	582	193	
XtIFNX12	KU594529	815074-814538	129063724-129064260	537	178	
XtIFNX13	KU594530	819613-819062	129068248-129068799	552	183	
XtIFNX14	KU594531	825344-824793	129073979-129074530	552	183	
XtIFNX15	KU594532	830073-829528	129078714-129079259	546	181	
XtIFNX16	KU594533	832104-831562	129080748-129081290	543	180	
XtIFNX17	KU594534	842896-842354	129091540-129092082	543	180	
XtIFNX18	KU594535	847552-847190	129096376-129096738	363	120	Partial orf
XtIFNX19	KU594536	855056-854505	129103691-129104242	552	183	
XtIFNX20	KU594537	859774-859220	129108406-129108960	555	184	
XtIFNX21	KU594538	868478-867933	129117119-129117664	546	181	
XtIFNX22	KU594539	870499-869957	129119143-129119685	543	180	
XtIFNX23	KU594540	874399-873857	129123043-129123585	543	180	
XtIFNX24	KU594541	883006-882452	129131638-129132192	555	184	
XtIFNX33	KU594550	785676-785112	129034240-129034871	612	203	
XtIFNX34	KU594551	788786-88212	129037329-129037927	564	187	
XtIFNX35	KU594552	798628-98054	129044371-129044996	600	199	
XtIFNX36	KU594553	756275-756275	129047237-129047814	543	180	
		Scaffold NW_004668804.1				
XtIFNX25	KU594542	8-331	129313251-129313579	329	109	Partial orf

XtIFNX26	KU594543	5550-6101	129318798-129319349	552	183	
XtIFNX27	KU594544	12403-12954	129325651-129326202	552	183	
XtIFNX28	KU594545	38405-38665	129351655-129351913	259	85	Partial orf
XtIFNX29	KU594546	41047-41511	129354295-129354759	465	154	
XtIFNX30	KU594547	43025-43480	129356273-129356728	456	151	
XtIFNX31	KU594548	55728-56267	129368976-129369515	540	179	
XtIFNX32	KU594549	59780-60331	129373028-129373579	552	183	
			Chr:08			
XtIFNL1	KU594554		60247518-60249466	459	152	
XtIFNL2	KU594555		60588080-60589959	612	203	
XtIFNL3	KU594556		60593518-60595131	618	179	
XtIFNL4	KU594557		60599038-60599488	444	147	
XtIFNL5	KU594558		60604364-60606214	540	179	
XtIFNL6	KU594559		60610432-60613216	540	179	
XtIFNLX1	KU594560	25555557-25556186 ***(Scaffold NW_004668234.1)***	99398436-99399065 ***(Chr:03)***	630	209	
Sub-total:	***50***					
X. laevis (9.1)			***Chr:9_10S (Assembly 9.1)***			
XaIFN1	KU594561		7980066-7983636	555	184	
XaIFN2	KU594562		7992970-7998176	642	213	
XaIFN3	KU594563		8002739-8011652	570	189	
XaIFN4	KU594564		8014967-8018581	594	197	
XaIFN5	KU594565		8023105-8025478	513	170	
XaIFN6	KU594566		8952158-8954550	495	164	
XaIFN7	KU594567		8961985-8964488	570	189	
			Scaffold 20			
XaIFNX1	KU594568		1400048-1399558	456	151	
XaIFNX2	KU594569		1402008-1402478	471	156	
XaIFNX3	KU594570		1403169-1403812	363	120	
XaIFNX4	KU594571		1413340-1412762	579	192	
XaIFNX5	KU594572		1428759-1428181	579	192	
XaIFNX6	KU594573		1439656-1440105	450	149	
XaIFNX7	KU594574		1446862-1446311	552	183	
XaIFNX8	KU594575		1447904-1449087	453	150	
XaIFNX9	KU594576		1449724-1450296	573	190	
XaIFNX10	KU594577		1456506-1457478	507	163	
XaIFNX11	KU594578		1460497-1461841	438	145	
XaIFNX12	KU594579		1465272-1465856	585	194	
XaIFNX13	KU594580		1497515-1498066	452	183	
XaIFNX14	KU594581		1499470-1499829	552	119	

XaIFNX15	KU594582		1500508-1501264	360	183	
XaIFNX16	KU594583		1502666-1503122	552	134	
XaIFNX17	KU594584		1511532-1513509	405	187	
XaIFNX18	KU594585		1496274-1496725	564	119	
			Chr:03L			
XaIFNX19	KU594586		132542751-132544220	567	175	
XaIFNX20	KU594587		132546560-132547144	585	194	
XaIFNX21	KU594588		132582941-132583929	717	238	
XaIFNX22	KU594589		132627459-132627671	654	217	
			Chr:08S			
XaIFNL1	KU594590		84913525-84915538	540	179	
			Chr:08L			
XaIFNL2	KU594591		59460764-59463028	612	203	
XaIFNL3	KU594592		59467262-59468860	540	179	
XaIFNL4	KU594593		59474415-59477003	540	179	
XaIFNL5	KU594594		59482333-59483889	540	179	
XaIFNL6	KU594595		59488691-59490331	459	152	
XaIFNL7	KU594596		59501405-59502544	393	116	
XaIFNL8	KU594597		59511519-59512760	591	196	
XaIFNL9	KU594598		59517290-59519662	540	179	
			Chr:03L			
XaIFNLX1	KU594599		99820872-99821423	552	183	
XaIFNLX2	KU594600		99830877-99831506	630	209	
Sub-Total:	***40***					
**Total:**	**90**					

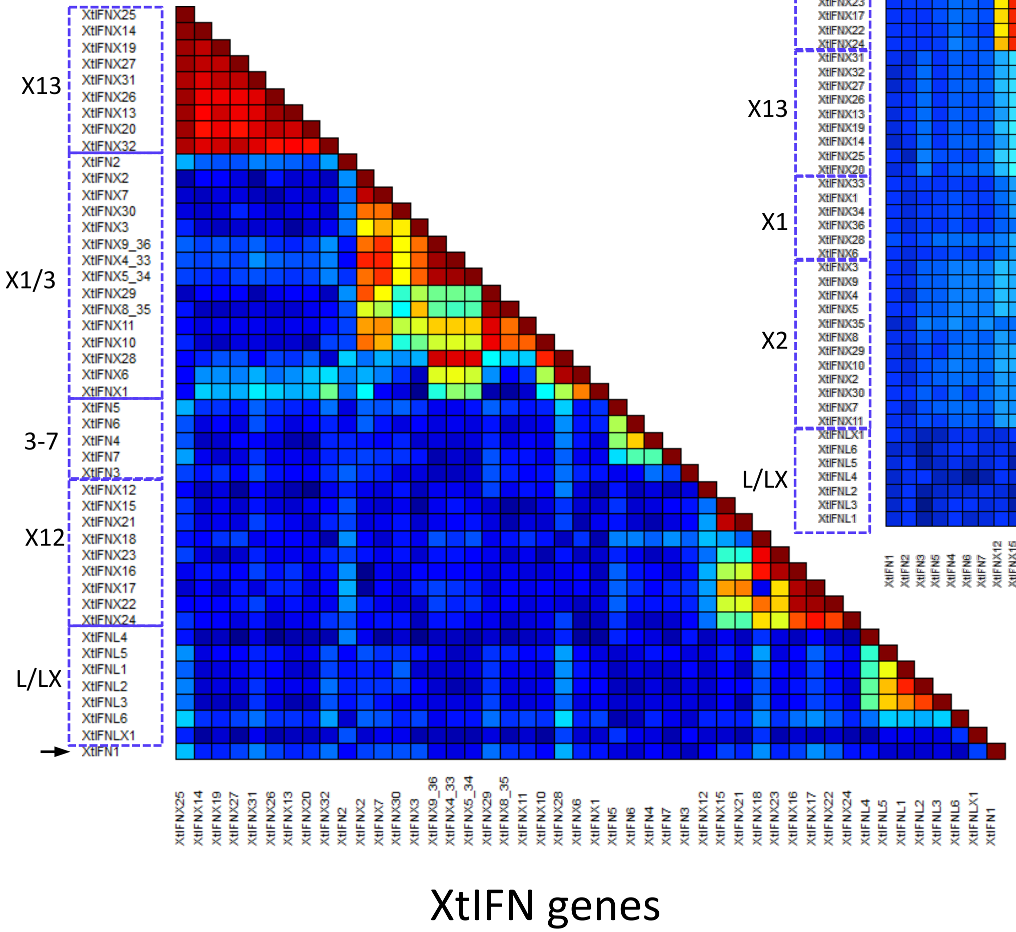

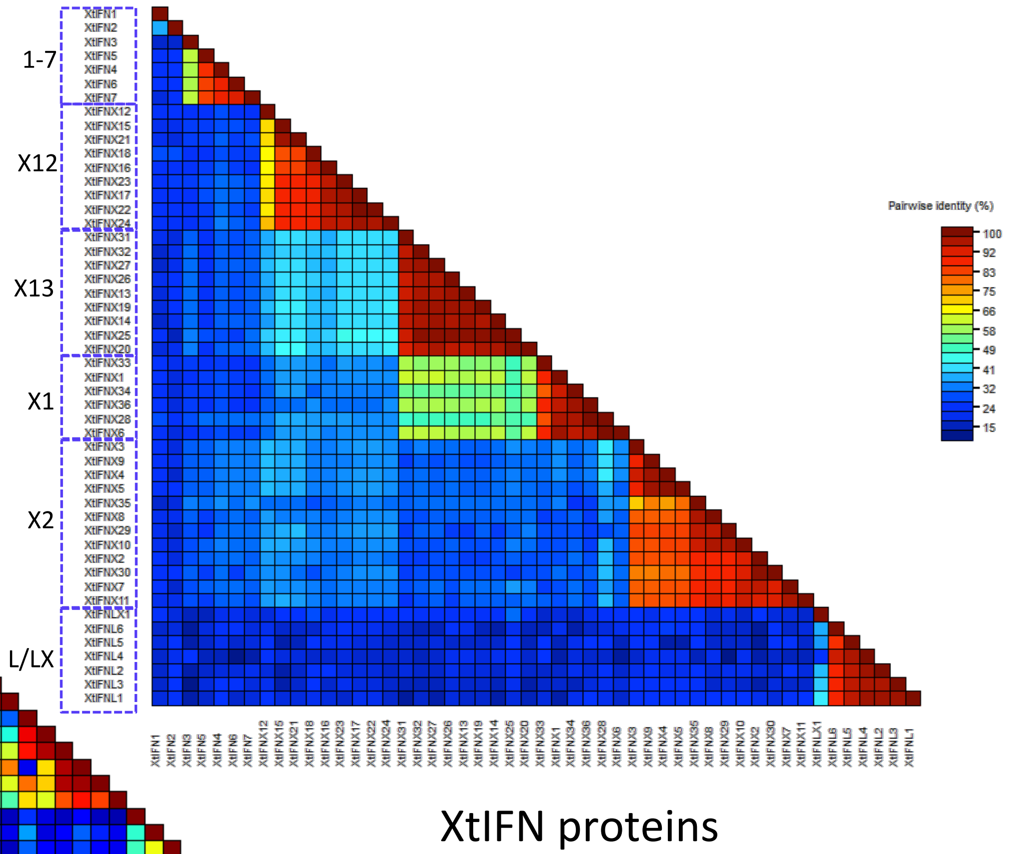

Figure S1. Pairwise identity (%) plots among protein (top right) and gene (bottom left) sequences of IFNs in *X. tropicalis*. The comparison and plot drawing were performed using a SDT program. Molecular subgroups of IFN complex in *X. tropicalis* were clustered based on the sequence identity, in particular of protein sequences, which generally show >60% pairwise identity among sequences within a subgroup. Noting that the gene sequences containing tentative 5'-promoter and 3'-untranslated regions (UTR) have more rapid diversification (bottom left), indicating further divergence pertinent to epigenetic regulation than genetic coding only.

Figure S2. Molecular subgroups of IFN complex in *X. tropicalis (*XtIFNs*)*. (A) The evolutionary analyses were conducted in MEGA6, and the tree was inferred by using the Maximum Likelihood method based on the Poisson correction model [1]. The percentage of trees in which the associated taxa clustered together is shown next to the branches. Subgroups were based on their phylogenic relationship at the molecular level. (B) The signal peptides of XtIFN precursors were examined using PrediSi (http://www.predisi.de) to determine the secretory potency of relevant IFN mature peptides, indicating the evolution of intracellular IFNs (pointed by arrows, Signal peptide prediction score 0-0.5) in each subgroup, particularly of intronless IFNs.

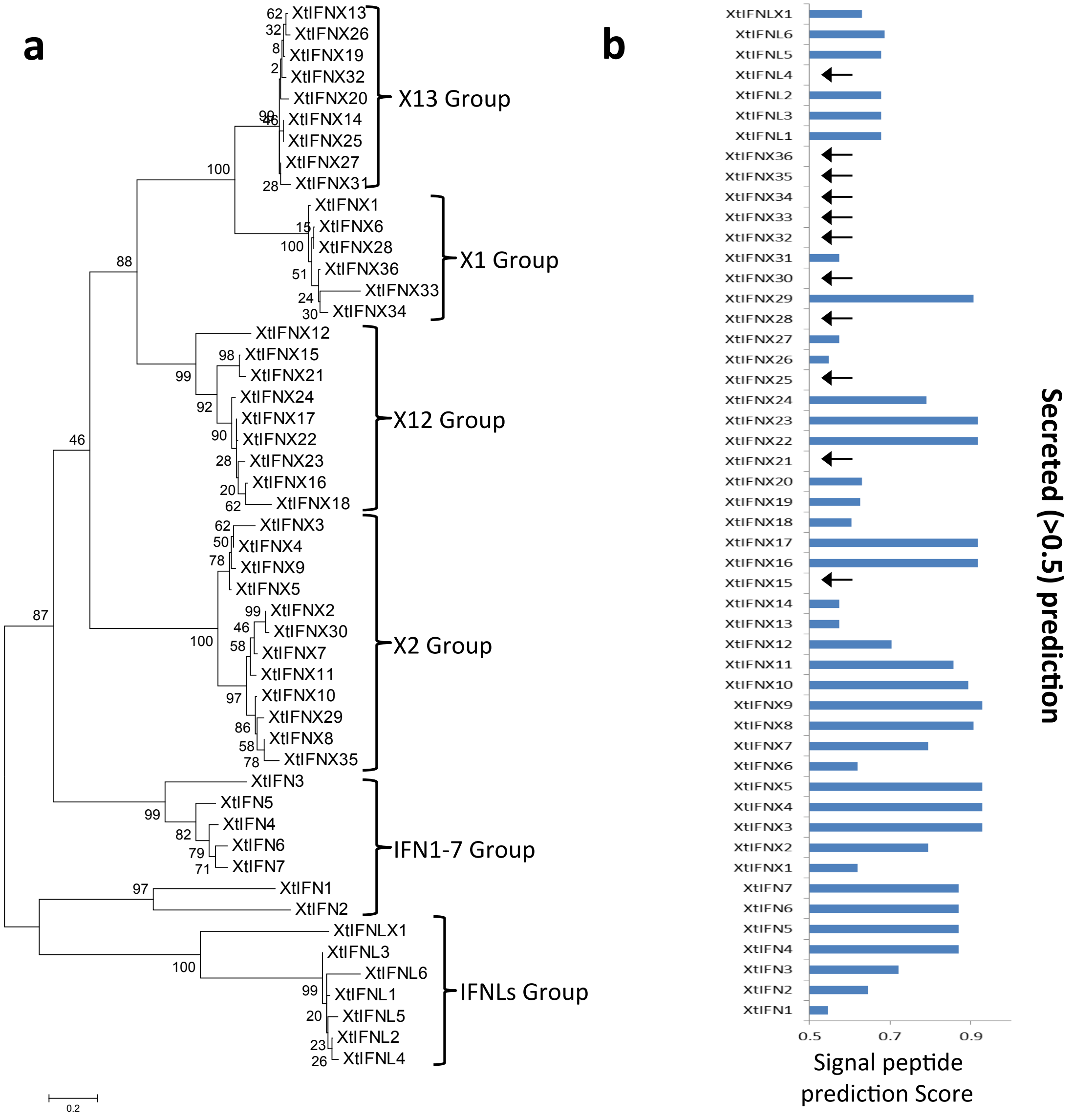

Supplemental Figure-S3a

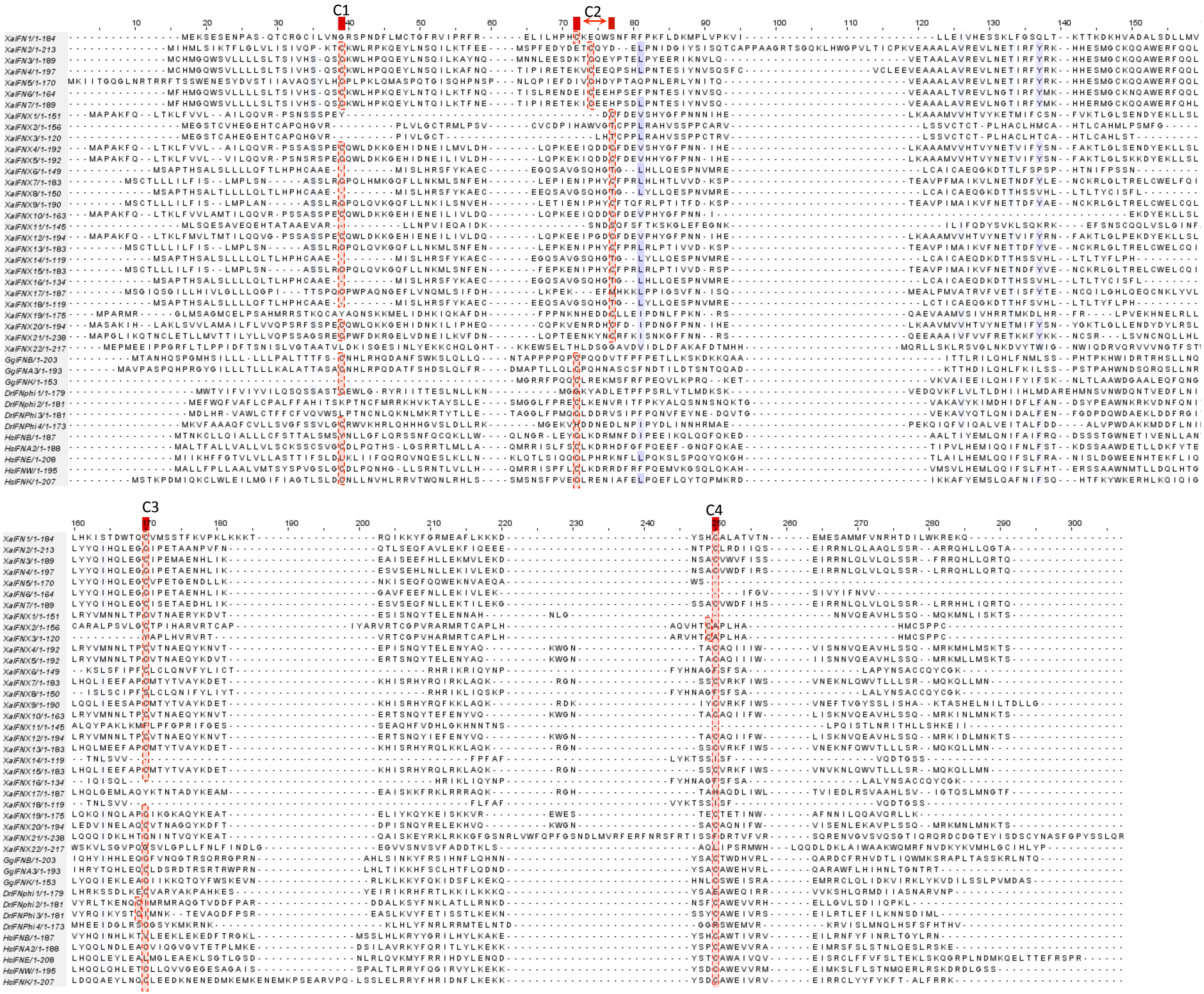

Supplemental Figure-S3b

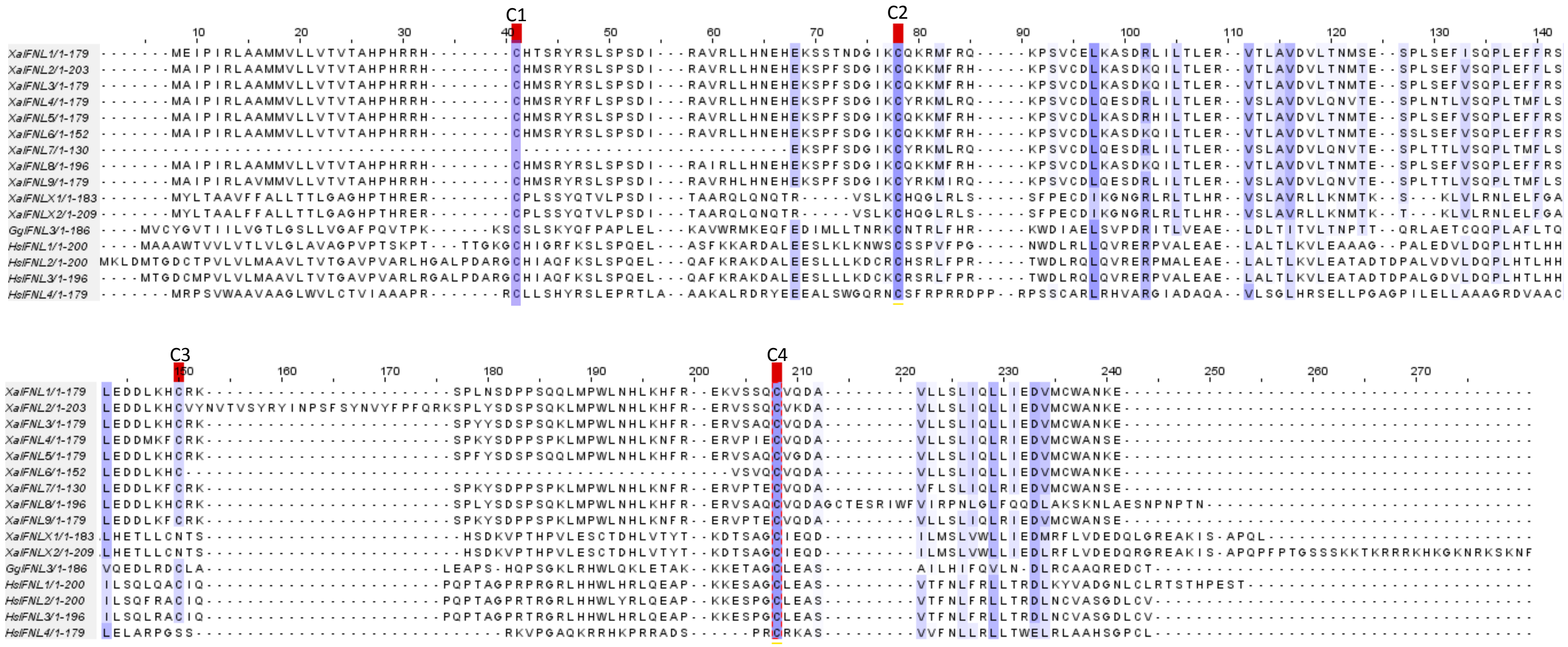

Supplemental Figure-S3c

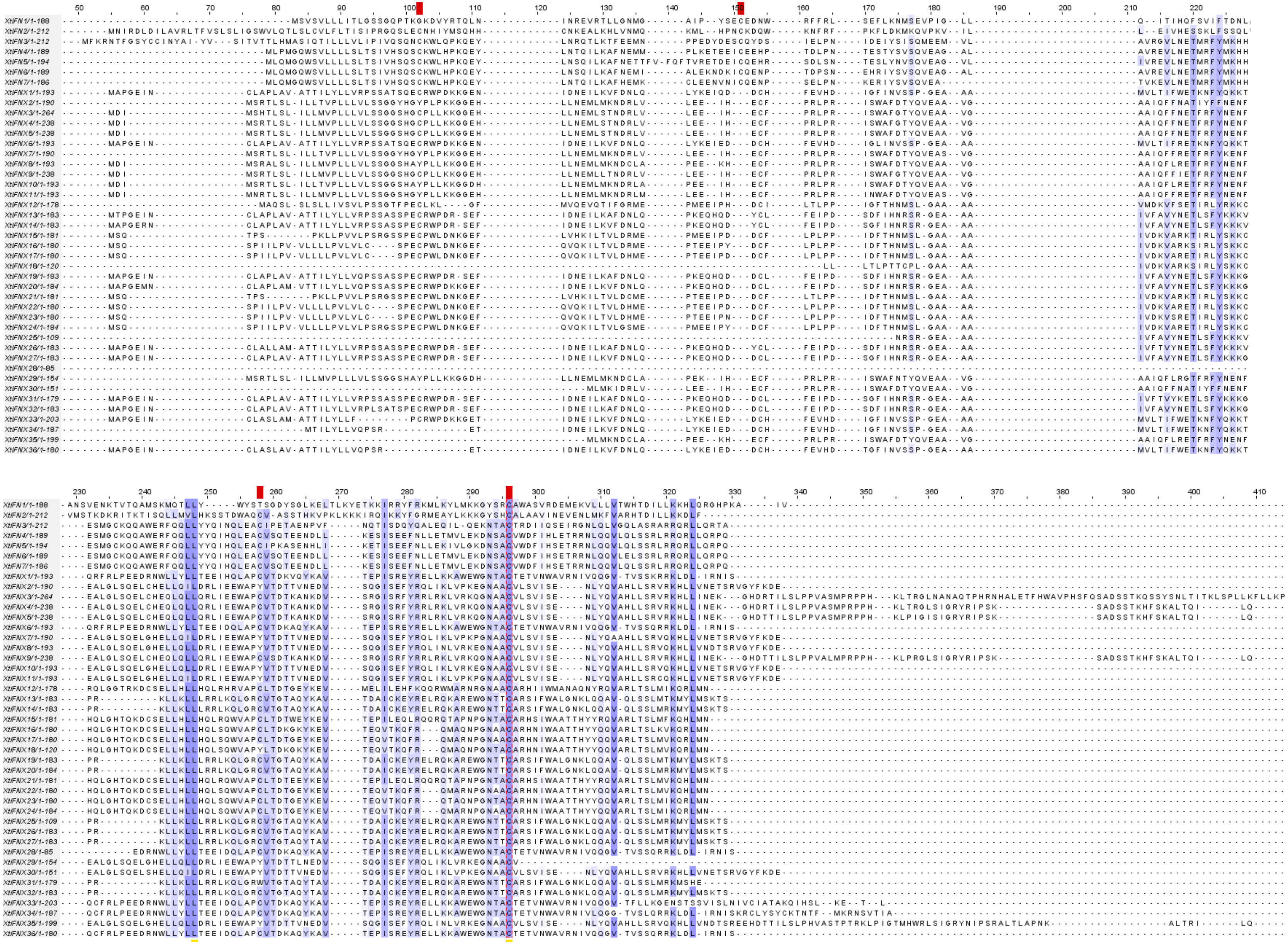

Supplemental Figure-S3d

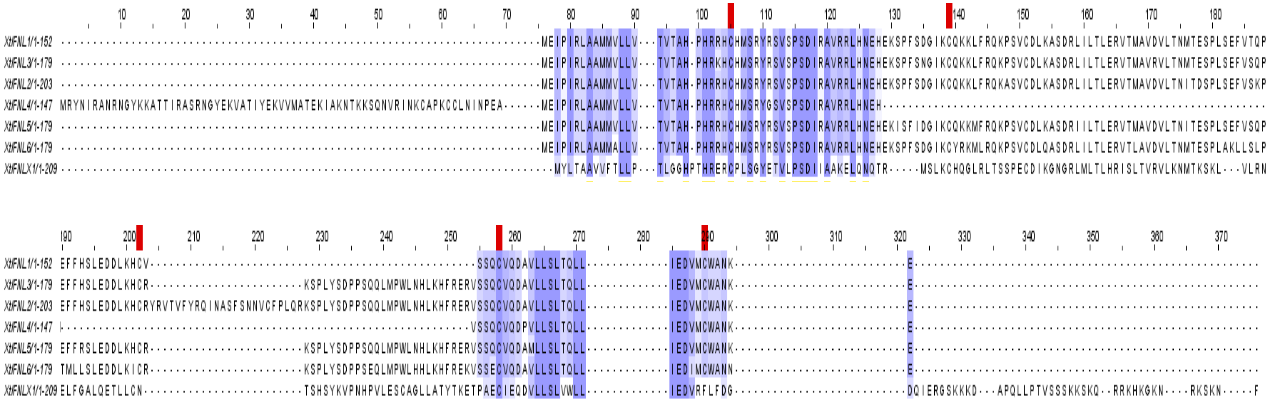

Figure S3. (A) Multiple sequence alignment of type I IFNs peptides (both intron-containing, XaIFNs, and intronless, XaIFNXs) in *X. laevis*. Frog IFN sequences, together with homologs from zebrafish (DrIFNs), chicken (GgIFNs), and humans (HsIFNs) were aligned using a MUSCLE program through the EMBL-EBI port (http://www.ebi.ac.uk/), and visualized with a Jalview program. The conserved residues, in particular, four cysteine residues were highlighted. (C) Multiple sequence alignment of type I IFNs peptides (both intron-containing, XtIFNs, and intronless, XtIFNXs) in *X. tropicalis*. Frog IFN sequences, together with homologs from zebrafish (DrIFNs), chicken (GgIFNs), and humans (HsIFNs) were aligned using a MUSCLE program through the EMBL-EBI port (http://www.ebi.ac.uk/), and visualized with a Jalview program. The conserved residues, in particular, four cysteine residues were highlighted. (D) Multiple sequence alignment of type III IFNs peptides (both intron-containing, XtIFNLs, and intronless, XtIFNLXs) in *X. tropicalis*. Frog IFN sequences, together with homologs from chicken (GgIFNL), and humans (HsIFNLs) were aligned using a MUSCLE program through the EMBL-EBI port (http://www.ebi.ac.uk/), and visualized with a Jalview program. The conserved residues, in particular, four cysteine residues were highlighted.

Putative proximal promoter region

-1.0 -0.9 -0.8 -0.7 -0.6 -0.5 -0.4 -0.3 -0.2 -0.1 0.0 TSS/ATG

10	9	8	7	6	5	4	3	2	1	0	
●	●●		●	●	●	●	◊●●	●●		●●●	XtIFN1
	●●	●●●	◊●●	●●●	●□	●	●○	●●○	●□	●	XtIFN4
	●	●●	◊□	◊●●		◊●○	●●		●●	●●	XtIFN5
	◊	●□	●	◊□	●□●	●●	●□	●●●	●●●	●○	XtIFNX15
◊	●□		●		●●●	●●	●●	●	●●●	●●	XtIFNX16
	◊	□	●		●●	●●●	●	●●●	●	●	XtIFNX17
◊	●□	●	◊□	●□●●	●	●□	●	○	●●●	●●	XtIFNX21
	◊	●□	□	●	●			●●	●●●	●□	XtIFNX22
	◊	□	●□	●●●	●●●	●●●	●□	●●●			XtIFNX23
◊	●□	□	●□		●●●	●	●○	●●●	●	●●●	XtIFNX24
●	●●●	●	●	●□●	●	●○	●●○	●●○	●	◊	XtIFNX26
	●	●●●	●	◊□●●	●●●	●●●	●		●●	□	XtIFNL3
●●●	●●	●●	●	●□●●	●◊	●●			●	●	XtIFNL5
●	●□	●●●	●	◊□●	●●			●●	●	●●●	XtIFNL6
	●□○	●◊	●□	●●●	●	●●	●●		●□●●	●□	XtIFNLX1

Figure S4. Schematic to categorize IFN genes based on regulatory elements predicted in their proximal promoter regions. The regulatory elements (and pertinent binding factors) in the ~1 kb proximal promoter regions was examined against both human/animal TFD Database using program Nsite (Version 5.2013, at http://www.softberry.com). Showings here include IFNs in *X. tropicalis* whose promoters containing at least one IFN- or virus-stimulated response elements (ISRE, PRDI, and/or STAT1/3 factors). The other groups of IFN genes, whose promoters do not contain these regulatory elements, and inclusive spread sheet of all predicted regulatory elements and relevant binding factors are listed in the Supplemental Excel Sheet xx. **Legend:** ○**,** containing such as GATA-1 regulating constitutive expression; ◊, containing IFN-stimulated response element (ISRE) and PRDI to interact with IRF, ISGF3 and STAT factors; □, containing cis-elements interacting with factors to mediate immune/inflammatory responses including C/EBP, NF-kB, NF-IL6, and p53; ●, containing cis-elements reacting with other factors significant in other developmental/physiological responses.

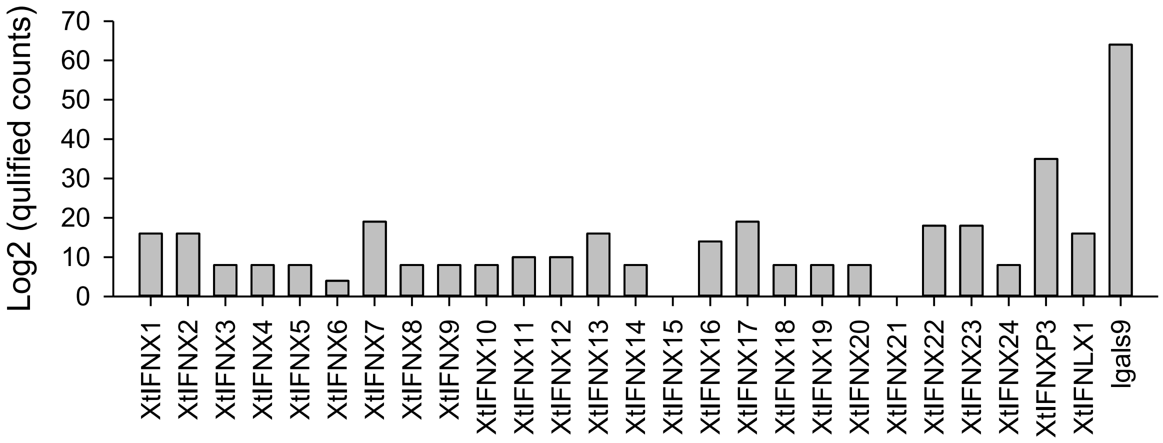

Figure 5S. Annotation of intronless XtIFN genes and mapping qualified RNA reads within the transcripts of most intronless XtIFNXs (XtIFNX1-24 and XtIFNLX1) through analysis of RNA-Seq data associated with the current genome assembly of *X. tropicalis (Silurana)* at NCBI (http://www.ncbi.nlm.nih.gov/genome/80, submitted by DOE Joint Genome Institute).

Supplemental Figure-S6a

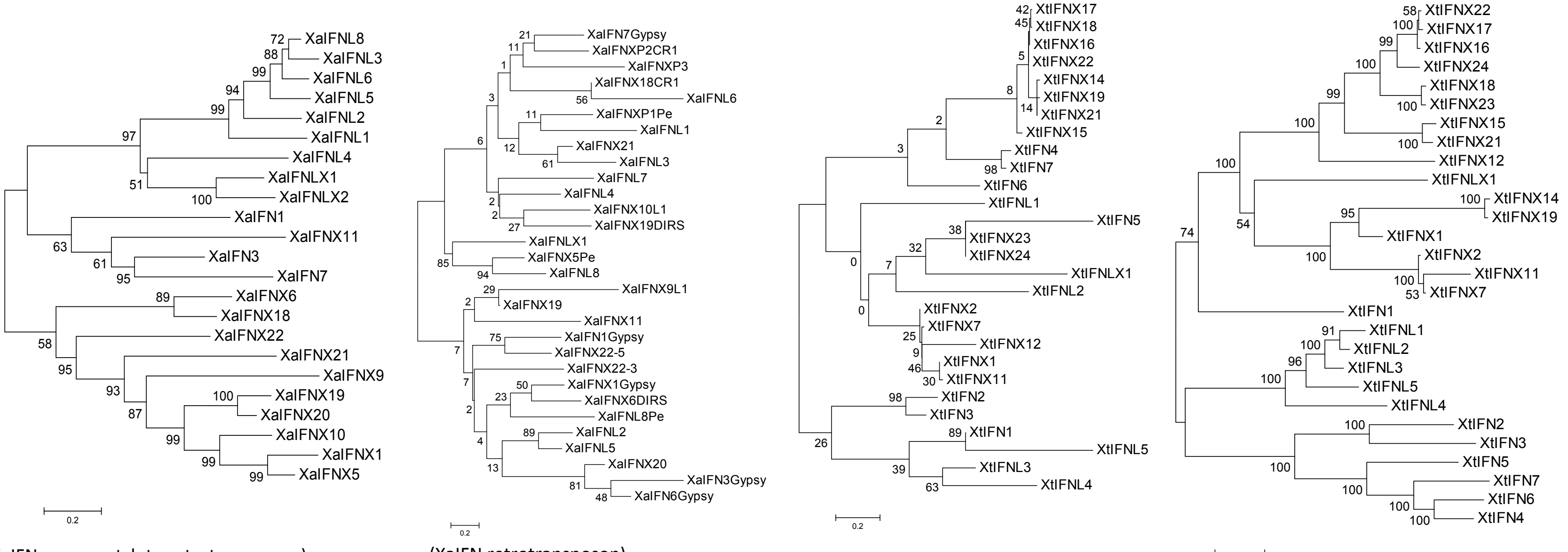

(XaIFN-gene-match to retrotransposon) (XaIFN retrotransposon) (XtIFN-gene retrotransposon) (XtIFN-gene match to retrotransposon)

Figure S6. (A) Topological comparison of phylogenic trees generated from IFN genes and the retrotransposons associated. (B) Topological comparison of phylogenic trees generated from IFN genes and their corresponding proximal promoters. The evolutionary history was inferred by using the Maximum Likelihood method based on the Tamura-Nei model. The percentage of trees in which the associated taxa clustered together is shown next to the branches. The tree is drawn to scale, with branch lengths measured in the number of substitutions per site. Evolutionary analyses were conducted in MEGA6. (B) Topological comparison of phylogenic trees generated from IFN genes and their corresponding proximal promoters. The evolutionary history was inferred by using the Maximum Likelihood method based on the Tamura-Nei model [1]. The percentage of trees in which the associated taxa clustered together is shown next to the branches. The tree is drawn to scale, with branch lengths measured in the number of substitutions per site. Evolutionary analyses were conducted in MEGA6 [2].

Supplemental Figure-S6b

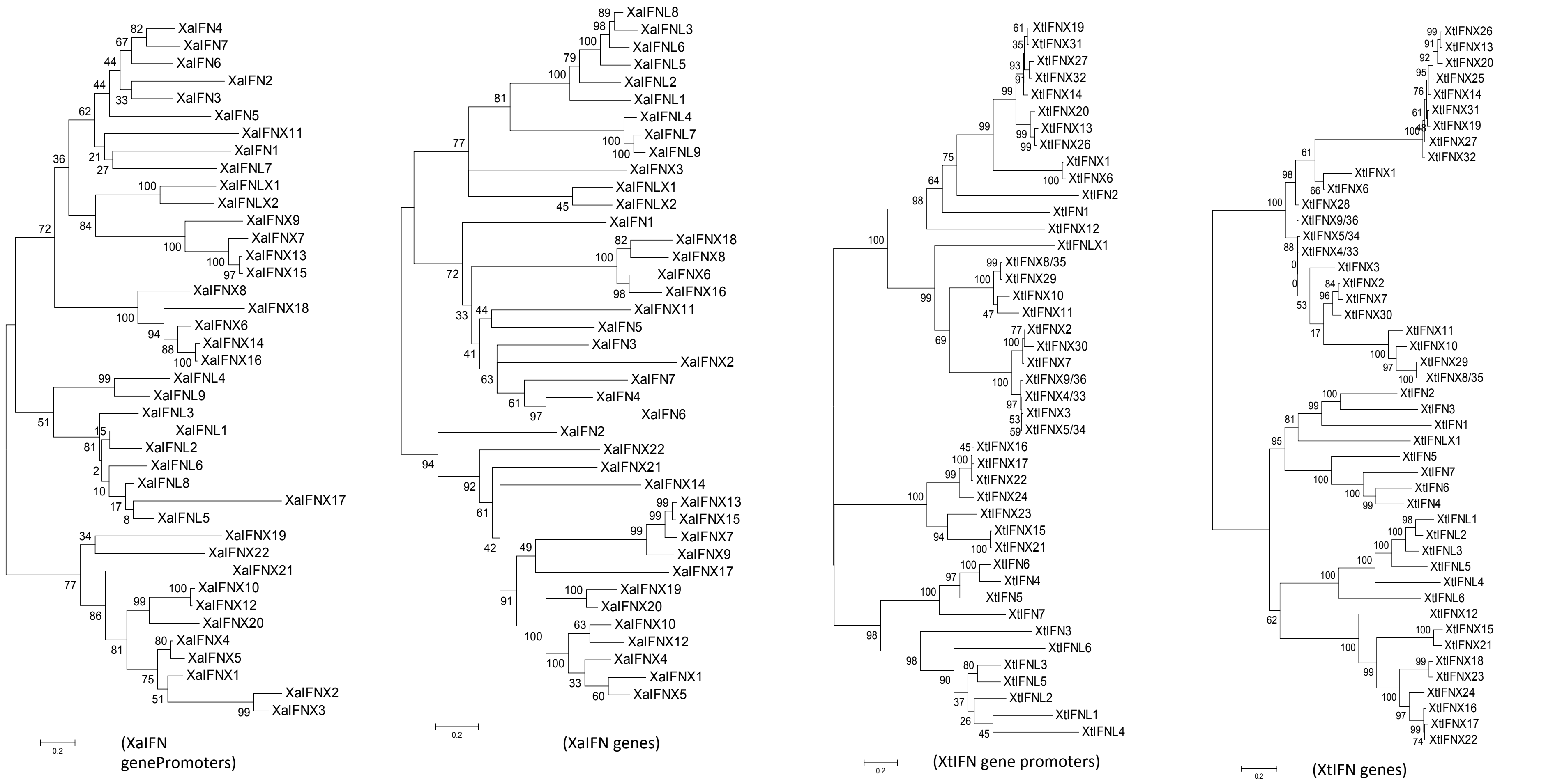

Figure S7. Typical influenza viruses (IAV) subtypes from swine have higher infectivity than those from humans and other animal species in the frog kidney cells. **(A)** Six IAV subtypes from swine, human, avian, and equine, were used to infect frog A6 cells at different doses (5, 10, and 20 MOI) and the virus infectivity was quantified by cytopathic effect (CPE) using a crystal violet staining procedure. All three tested swine IAV strains showed higher infectivity than the isolates from humans and other animal species; in particular, the TX98 swine IAV seems well adapted to the frog cells causing significant infections at various testing conditions. **(B-G)** Photomicrographs of frog A6 cells infected by IAV from different animal species to show the significant infections by the swine TX98 and vH1N2 viruses at 3 MOI. The brown spots (arrowheads) in the images are immunohistochemical labeling of IAV using a mAb against influenza nucleoprotein (NP) (H16-L10-4R5, ATCC, HB-65™). The data and micrographs represent results of four independent tests with similar observation.

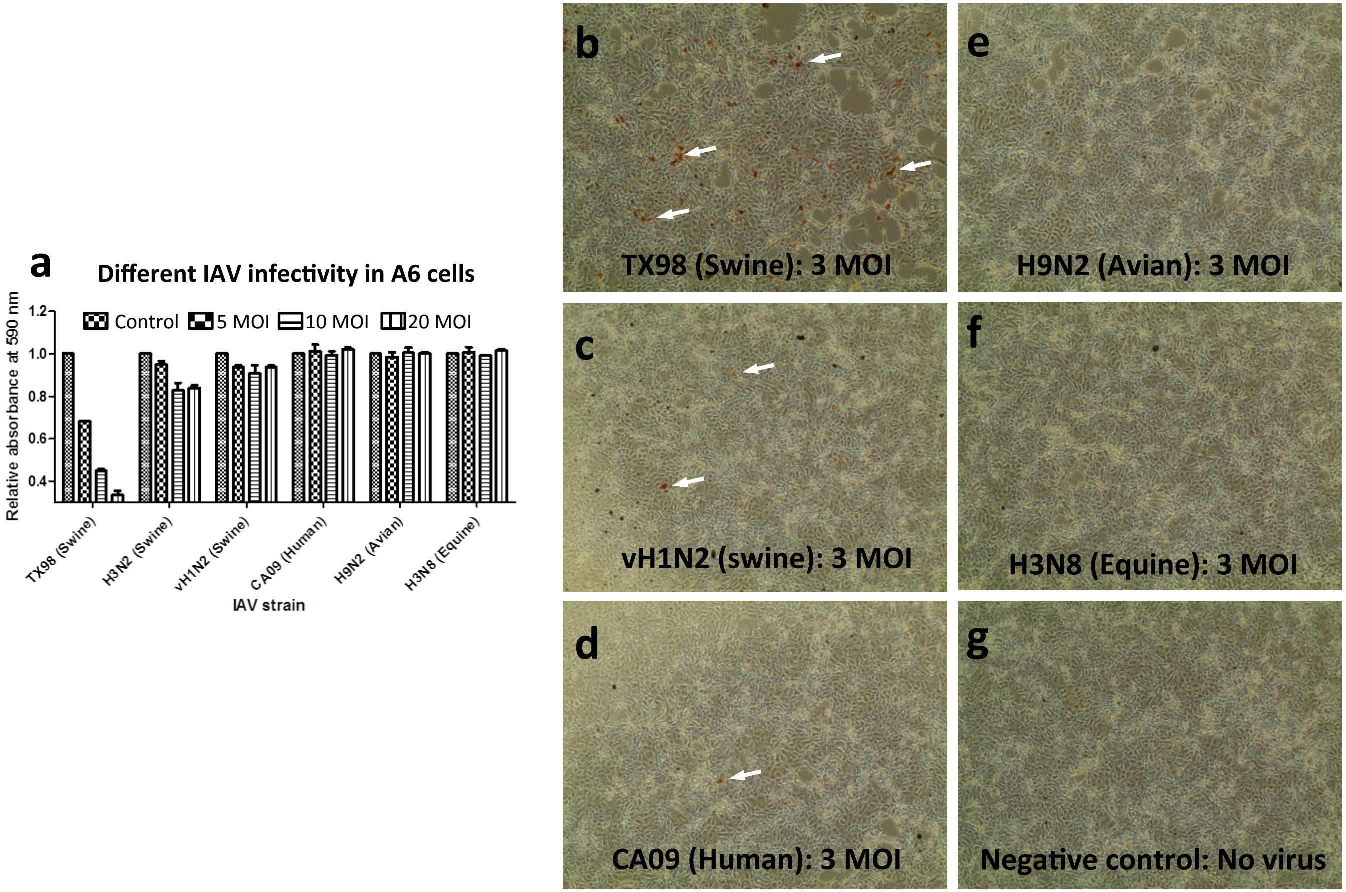

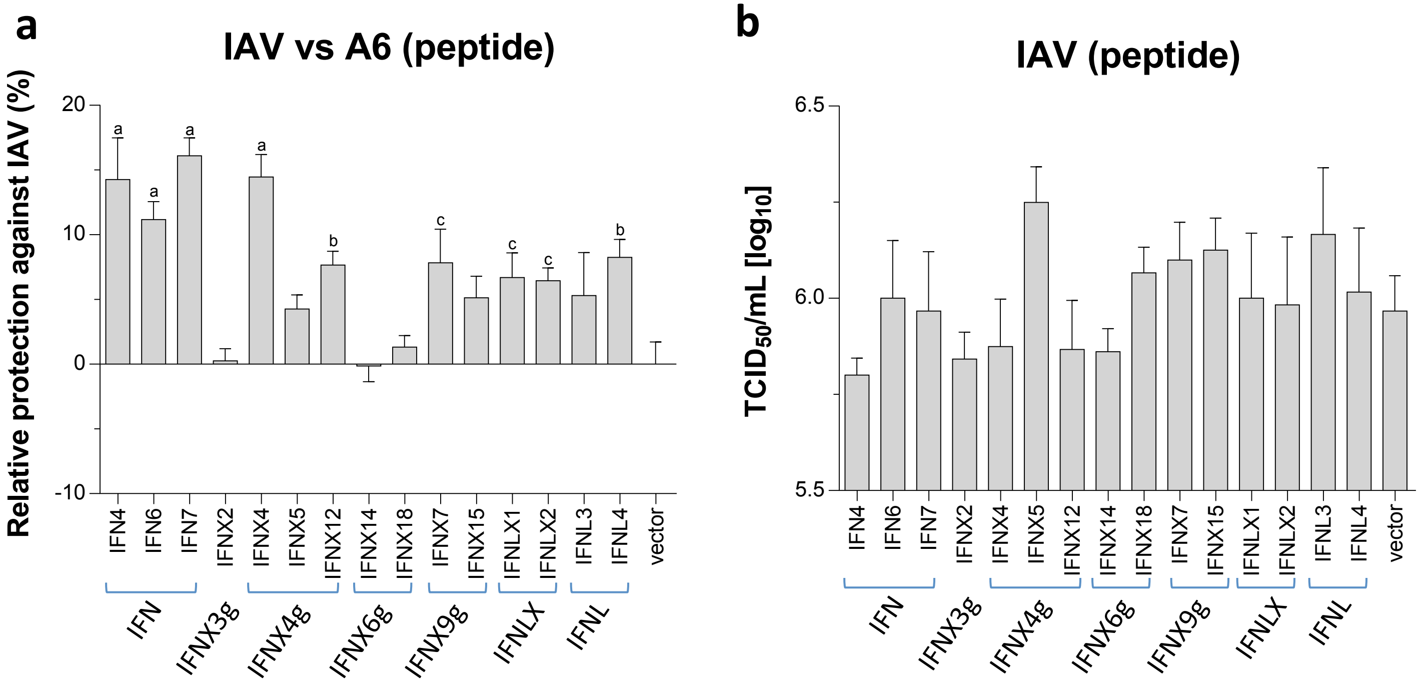

Figure S8. Antiviral activity of *X. laevis* IFN peptides (IFNs) in amphibian kidney cells (A6). (**A**) Confluent frog A6 kidney cells were treated overexpressed IFN peptides for 24 h, then were infected with the swine TX98 virus at MOI of 5 for 48 h. The protection of IFNs from the viral or infections was then quantified at 2 days post infection using a crystal violet staining procedure. (**B**) The culture supernatants in (A) were collected for virus titration using an endpoint dilution assay to define 50% tissue culture infective dose ($TCID_{50}$) in MDCK cells. The data represent results of two independent experiments. Data are means ± SE; n=3 replicates, a, b, and c: p<0.001, 0.01 and 0.05, respectively to the control. Primers for RT-PCR detection and cloning were listed in the Supplemental Excel Sheet 9.

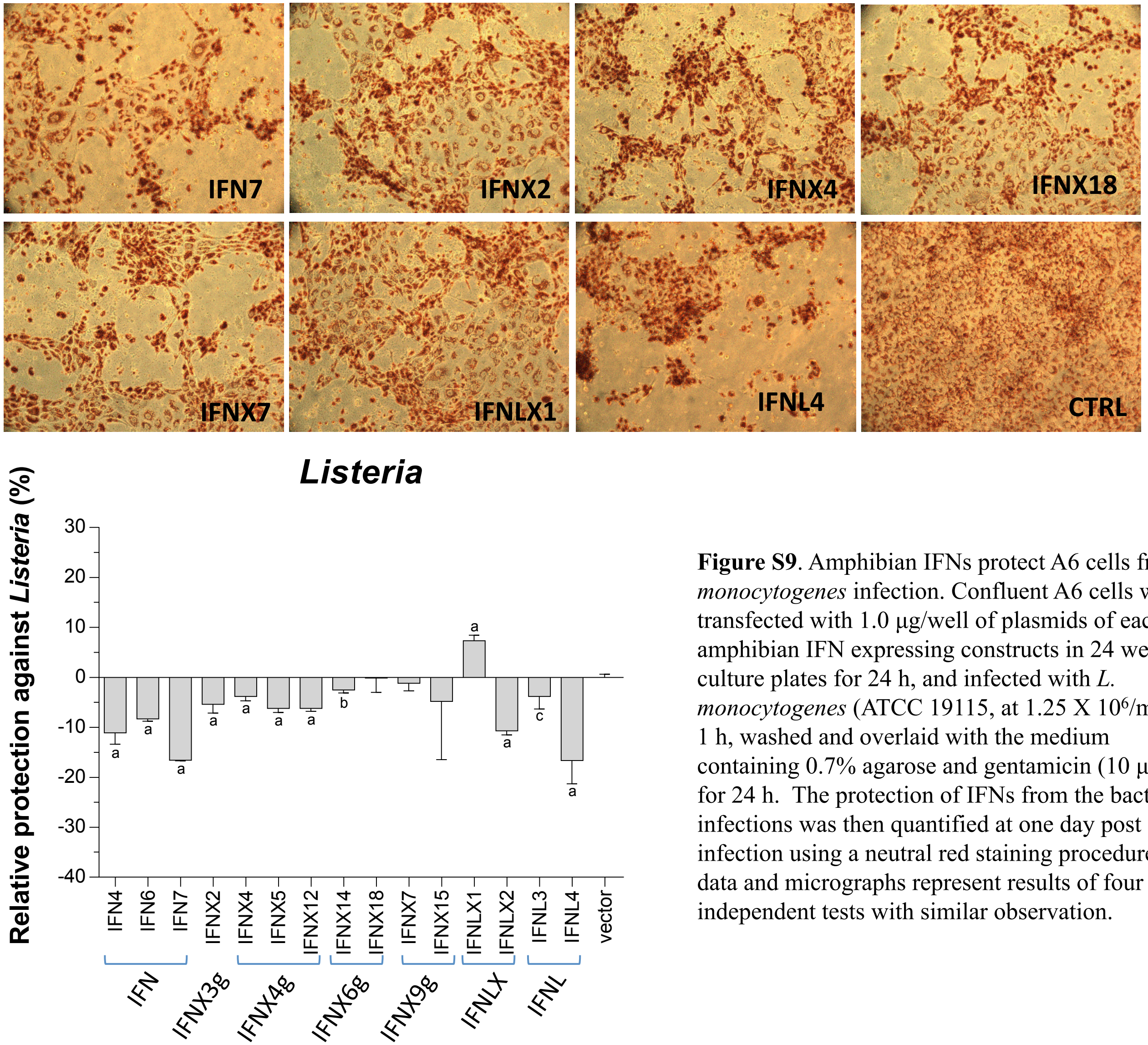

Figure S9. Amphibian IFNs protect A6 cells from *L. monocytogenes* infection. Confluent A6 cells were transfected with 1.0 µg/well of plasmids of each amphibian IFN expressing constructs in 24 well cell culture plates for 24 h, and infected with *L. monocytogenes* (ATCC 19115, at 1.25 X 10^6/ml) for 1 h, washed and overlaid with the medium containing 0.7% agarose and gentamicin (10 µg/ml) for 24 h. The protection of IFNs from the bacterial infections was then quantified at one day post infection using a neutral red staining procedure. The data and micrographs represent results of four independent tests with similar observation.
